# Integrative phenotyping framework (iPF): integrative clustering of multiple omics data identifies novel lung disease subphenotypes

**DOI:** 10.1186/s12864-015-2170-4

**Published:** 2015-11-11

**Authors:** SungHwan Kim, Jose D. Herazo-Maya, Dongwan D. Kang, Brenda M. Juan-Guardela, John Tedrow, Fernando J. Martinez, Frank C. Sciurba, George C. Tseng, Naftali Kaminski

**Affiliations:** Department of Internal Medicine (Pulmonary, Critical Care and Sleep Medicine), Yale School of Medicine, New Haven, CT 06520 USA; Department of Biostatistics, University of Pittsburgh, Pittsburgh, PA 15261 USA; Department of Medicine, University of Pittsburgh, Pittsburgh, PA 15261 USA; Department of Medicine, Weill Cornell Medical College, New York, NY 15261 USA; Department of Human Genetics, University of Pittsburgh, Pittsburgh, PA 15261 USA; Genomics Division, Lawrence Berkeley National Laboratory, Berkeley, CA 94720 USA; Department of Statistics, Korea University, Seoul, 5062 South Korea

**Keywords:** Cluster analysis, Genomics, Chronic lung disease, Integrative clustering

## Abstract

**Background:**

The increased multi-omics information on carefully phenotyped patients in studies of complex diseases requires novel methods for data integration. Unlike continuous intensity measurements from most omics data sets, phenome data contain clinical variables that are binary, ordinal and categorical.

**Results:**

In this paper we introduce an integrative phenotyping framework (iPF) for disease subtype discovery. A feature topology plot was developed for effective dimension reduction and visualization of multi-omics data. The approach is free of model assumption and robust to data noises or missingness. We developed a workflow to integrate homogeneous patient clustering from different omics data in an agglomerative manner and then visualized heterogeneous clustering of pairwise omics sources. We applied the framework to two batches of lung samples obtained from patients diagnosed with chronic obstructive lung disease (COPD) or interstitial lung disease (ILD) with well-characterized clinical (phenomic) data, mRNA and microRNA expression profiles. Application of iPF to the first training batch identified clusters of patients consisting of homogenous disease phenotypes as well as clusters with intermediate disease characteristics. Analysis of the second batch revealed a similar data structure, confirming the presence of intermediate clusters. Genes in the intermediate clusters were enriched with inflammatory and immune functional annotations, suggesting that they represent mechanistically distinct disease subphenotypes that may response to immunomodulatory therapies. The iPF software package and all source codes are publicly available.

**Conclusions:**

Identification of subclusters with distinct clinical and biomolecular characteristics suggests that integration of phenomic and other omics information could lead to identification of novel mechanism-based disease sub-phenotypes.

**Electronic supplementary material:**

The online version of this article (doi:10.1186/s12864-015-2170-4) contains supplementary material, which is available to authorized users.

## Background

Disease phenotyping refers to a procedure that specifies disease definition or diagnosis in terms of observable abnormal phenotypic characteristics that occur due to the interaction between genotypes and environmental effects. Traditionally, the two largest pulmonary disease phenotypes—obstructive and restrictive lung diseases—have been determined using physiological, radiological, or histopathological features. Our study focuses on the most common diseases representing these aforementioned phenotypes—COPD and ILD. COPD is a lung disease caused by the repeated exposure to a noxious agent resulting in irreversible airflow limitation. COPD is classified by the Global Initiative for Chronic Obstructive Lung Disease criteria in four major categories based on symptoms, airflow obstruction, and exacerbation history [1]. Similarly, the term Interstitial Lung Disease designates a loosely defined group of patients characterized by changes in the interstitium of the lung, causing pulmonary restriction and impaired gas exchange. This group includes: Idiopathic Pulmonary Fibrosis (IPF), Non Specific Interstitial Pneumonia (NSIP), Hypersensitivity Pneumonitis (HP), Cryptogenic Organizing Pneumonia (COP), Respiratory Bronchiolotis-associated Interstitial Lung Disease (RB-ILD), Collagen Vascular Disease—associated Interstitial Lung Disease (CVD-ILD), Desquamative Interstitial Pneumonia (DIP) and Acute Interstitial Pneumonia (AIP), among others.

Despite the advancement in phenotyping these two broad lung disease categories based on traditional methods, current clinical definitions and classifications of COPD or ILD often fail to accommodate the large number of patients with atypical features who typically fall into undefined categories [2]. Moreover, existing classifications do not reflect advances in high-throughput mRNA and miRNA expression techniques that may improve our understanding of the complexity of a given individual’s phenotype. In this paper, we refer to “phenome” as the collection of traditional disease phenotypes described above, which is in contrast to measurements from rapidly developing high-throughput omics techniques. The purpose of this paper is to provide a generalizable phenotyping procedure by combining phenome and other omics data (mRNA and miRNA expression in our example) for novel disease subtype discovery.

As the array and massively parallel sequencing costs keep dropping, omics data generation has increased at an unprecedented rate. Meaningful integration and presentation of the abundant information has led to new computational and statistical challenges. According to Tseng et al. (2012) [3], omics data integration contains two major categories: horizontal meta-analysis and vertical integrative analysis. In the former type of data integration, the same type of omics data sets (e.g. gene expression, GWAS or eQTL) are collected from different labs and aligned horizontally with gene features matched on the rows. The major purpose of the analysis is similar to traditional meta-analysis, repeated over all features on the genome for candidate biomarker or pathway detection. In the latter analysis, multi-layers of omics data (such as genotyping, gene expression, miRNA expression, methylation and mutations) are measured in a given patient cohort and integrative analyses are performed to understand the inter-omics disease mechanism and relationship. As an example, the Cancer Genome Atlas (TCGA) [4] contains multi-omics data for more than 10,000 patients, and spans more than 20 cancers. Vertical information integration of multi-layer omics data has gained increasing attention in the past few years in biomedical research [5–8]. Depending on biological purposes, many tools have been developed. A large collection of existing integrative applications utilize relatively naïve summary/comparative scores (e.g. correlations and signal-to-noise ratios) and visualization tools (e.g. heatmaps, scatter plots, volcano plots, box plots and survival curves) with minimal statistical information integration [9, 10] and several convenient packages or pipelines along this approach are available [11, 12]. Other advanced statistical and computational methods have been rapidly developed. For example, methods have been developed to integrate copy number variation or methylation with gene expression profiles [13, 14]. Dimension reduction methods including principal component analysis [15], partial least squares [16], and nonnegative matrix factorization [17] have been applied to identify homogeneous and heterogeneous patterns across multi-omics data. To identify novel disease subtypes, the Bayesian consensus clustering [18] and iCluster [19] are two recent powerful methods to combine multi-omics data. They are, however, limited by a few drawbacks that motivated the development of our new integrative phenotyping framework (iPF). First, both Bayesian consensus clustering and iCluster assume numerical (continuous) measurements in the multi-omics data but the complexity of phenome data that contains binary, ordinal and multi-class categorical data types is not addressed. Second, both methods are model-based and poor performance is expected if the distribution assumptions are violated. Finally, both methods lack visualization tools for further exploratory analysis. The proposed iPF described in this paper aims to fill these gaps. iPF incorporates automatic feature selection, dimension reduction, data smoothing and pattern visualization in the feature topology plot. Single-omic cluster analysis is applied to each omics data set and a workflow to encompass homogeneous and heterogeneous clustering information across omics data is performed. In our application to the COPD and ILD data, novel disease subphenotypes were characterized and validated by training and testing batches. Post-hoc functional analysis revealed important biological processes related to the disease subtypes that might lead to novel diagnosis or treatment strategy. All data sets and source code used in this paper are publicly available as an iPF R package (http://tsenglab.biostat.pitt.edu/software.htm).

## Results

### Overview of Integrative phenotyping framework (iPF)

**Fig. 1:**
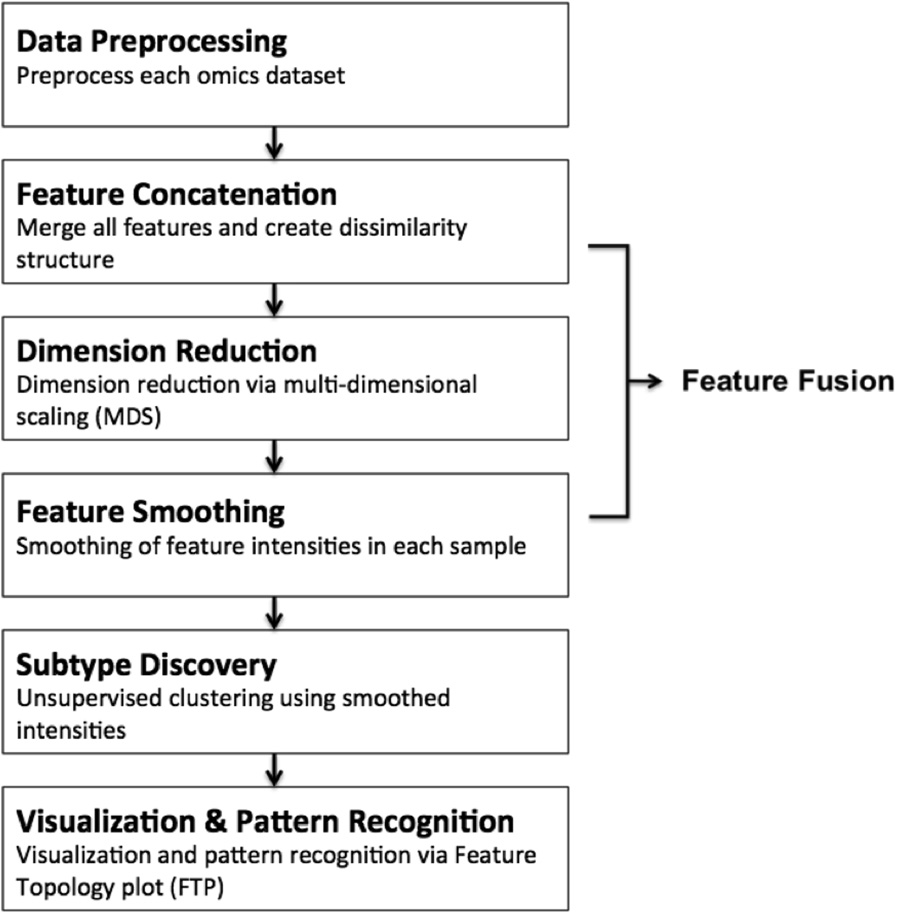
Flow chart of Integrative Phenotyping Framework (iPF): Integrative phenotyping framework (iPF) includes the following steps (1) Data preprocessing (2) Feature concatenation (3) Dimension reduction (4) Feature smoothing (5) Clustering for subtype discovery and visualization

**Fig. 2:**
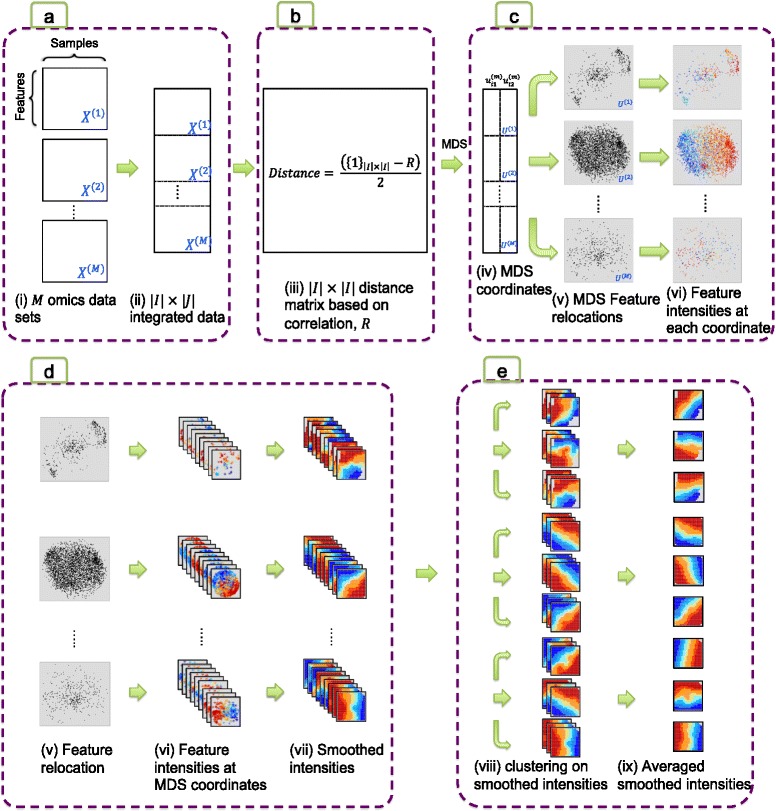
Overview of integrative clustering in integrative phenotyping framework (iPF): (**a**) Vertically combined multiple omics data sets (**b**) A distance matrix between any two features within and across omic data sets (**c**) Multidimensional scaling (MDS) mapping to a two-dimensional Euclidean space (**d**) Smoothed feature intensities in the reduced 2D space for each patient (**e**) Unsupervised clustering to identify potential disease subtypes and averaged feature intensities for representative plots of each cluster

**Fig. 3:**
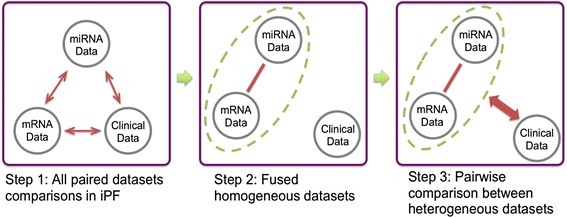
Graphical illustration of heterogeneous data sets comparison scheme in iPF. Step 1-2: Compare and combine all possible pairs of omics data sets until we produce homogenous clustering results. Step 3: Pairwise comparison until identifying any heterogeneous data sets

**Fig. 4:**
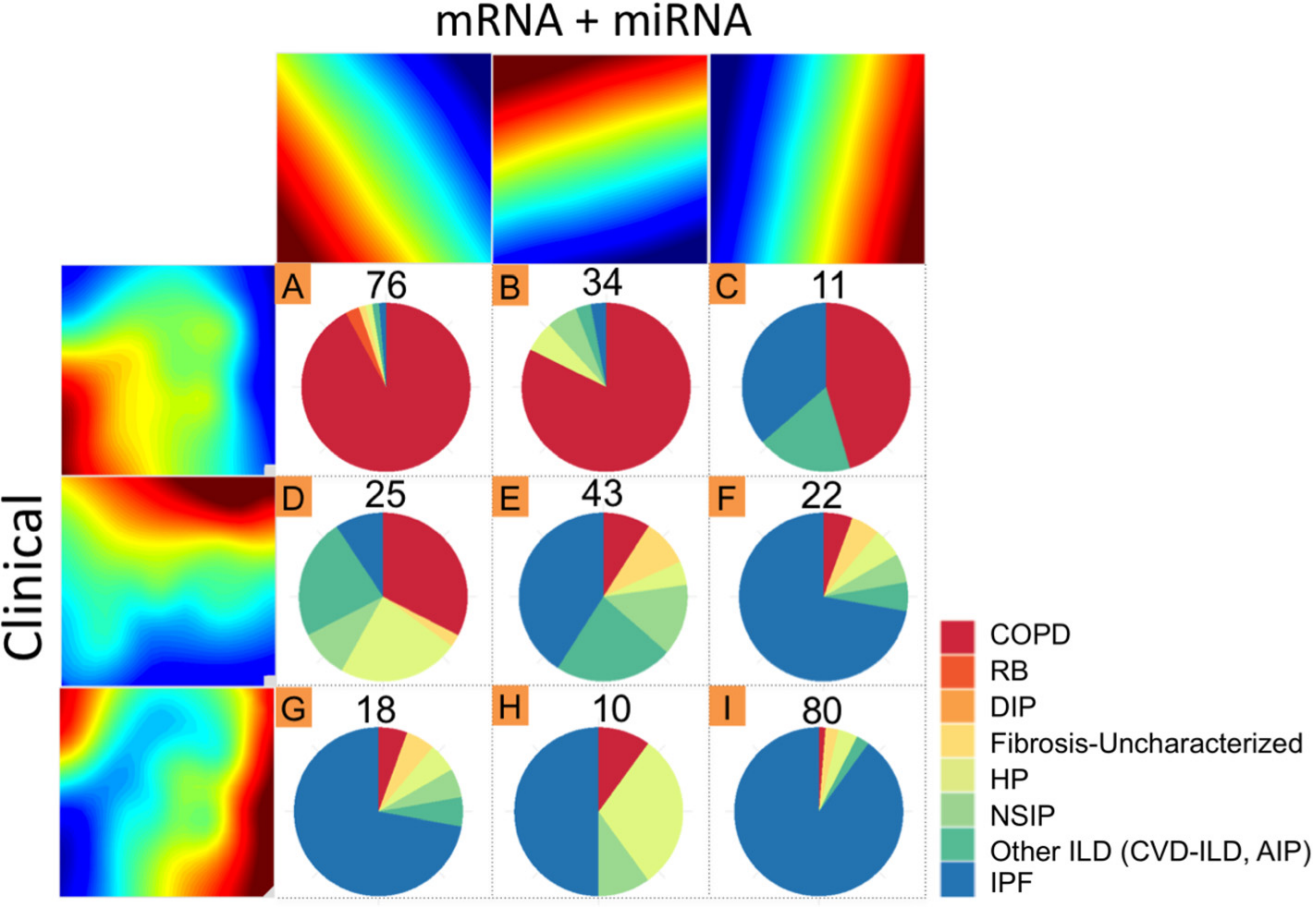
iPF clustering results for multiple omics data sets using all samples (Clinical, mRNA + miRNA). This figure shows three clusters are generated in each omics data source, and the feature topology plots for clusters from the first and the second omics data sources on the left and on the top, respectively

**Fig. 5:**
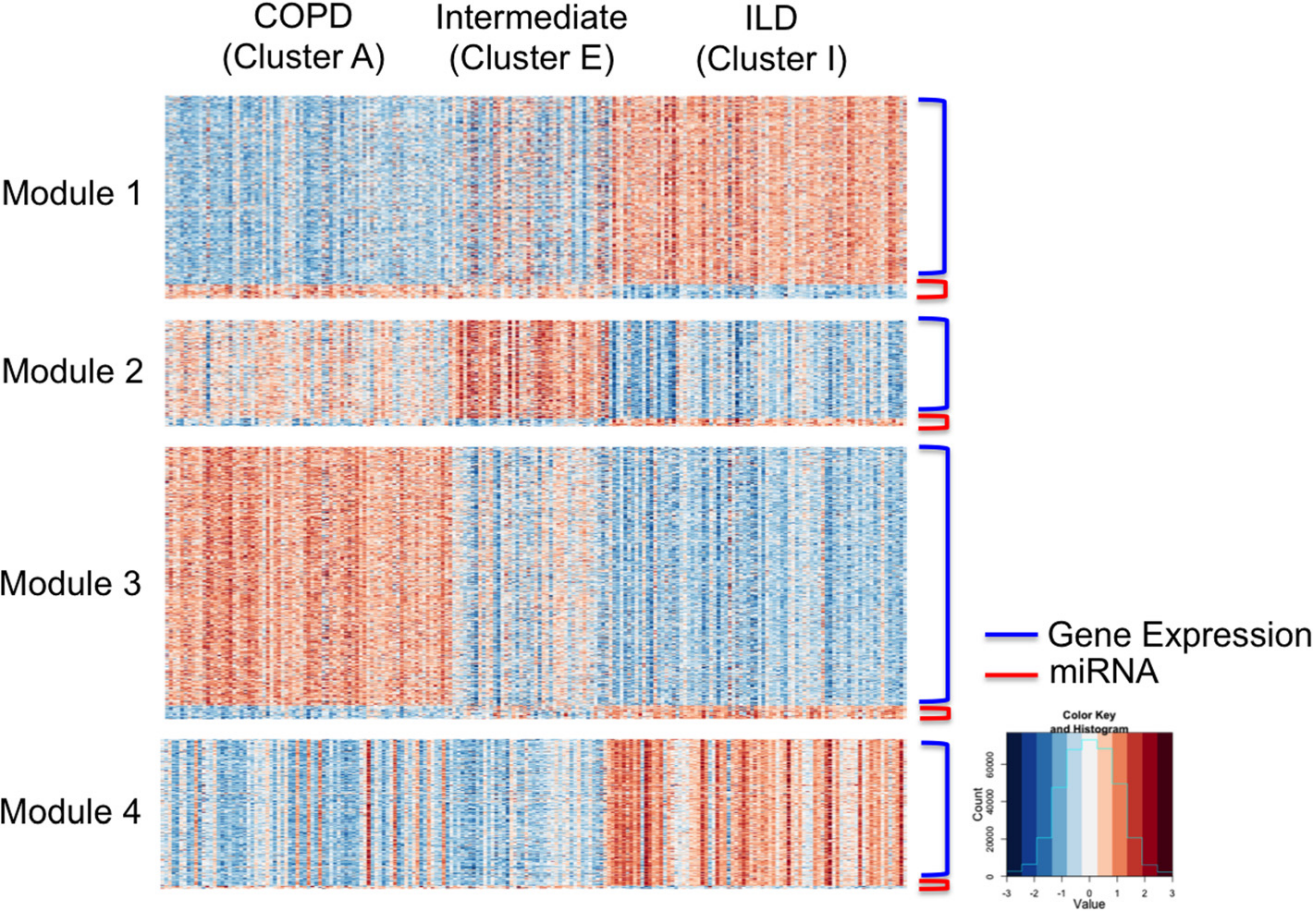
Heatmap for the four modules of gene expression and miRNA features which significantly differentiate three clusters (COPD, Intermediate, and ILD). We performed gene co-expression cluster analysis using partition around modoids (PAM) to identify four gene and miRNA modules. When perform clustering, the 88 miRNAs intensities are turned to the opposite direction (by multiplying the expression intensities by -1) to show that most miRNAs have inhibitory effects on mRNA expression

The integrative phenotyping framework (iPF) consists of the following four steps in the flowchart (Fig. 1): (1) Data pre-processing: Each omics data set is adequately pre-processed and normalized. Redundant (e.g. non-expressed and/or non-informative) features are separately eliminated in each omics data set; (2) Feature concatenation: Omics data sets are vertically combined as in Fig. 2a. A distance (dissimilarity) matrix between any two features within and across omics data sets is defined (Fig. 2b); (3) Dimension reduction: Multidimensional scaling (MDS) is applied to map all features to a two-dimensional Euclidean space for dimension reduction (Fig. 2c); (4) Feature smoothing: Feature intensities are smoothed in the reduced 2D space for each patient (Fig. 2d); (5) Clustering for subtype discovery and visualization: Unsupervised clustering analysis is performed to identify potential disease subtypes, and feature intensities within each cluster are averaged to generate representative plots for each cluster (Fig. 2e). The resulting contour plots are referred to as “feature topology plots (FTP)”, hereafter. Details of the iPF framework are presented in the method section and supporting information.

Different omics data may contribute to similar (homogeneous) or distinct (heterogeneous) disease subtype definition. For example, iCluster vertically aligns multi-omics data and performs latent variable decomposition. It implicitly assumes that different omics data contribute to one final and common disease subtype definition. The Bayesian consensus clustering method alternatively models common and distinct patient subtypes from different omics data. In our framework, we perform pairwise agglomerative merging strategy when a pair of omics data sets present “homogeneous” clustering results. For example, when three omics data sets (mRNA, microRNA (miRNA) and clinical) are available in Fig. 3, all pairs of omics data sets are compared. If mRNA and miRNA generate similar clustering results while the other two pairs do not, we merge mRNA and miRNA. Finally, we compare the clustering results of mRNA + miRNA versus clinical. This strategy can be generalized to combining three or more omics data sets and systematically but dynamically investigate clusters identified in different omics data. To determine homogeneity or heterogeneity of clustering results, Fig. 4 shows the feature topology plots for clusters from the first and the second omics data sources on the left and on the top (three clusters in each omics data source). The number of overlapped patients in the 3 × 3 table provides evidence of homogeneity (majority of patients are on the diagonal; for example, mRNA vs miRNA in Additional file 1: Figure S8(a)) or heterogeneity (existence of clusters off-diagonal as shown in Additional file 1: Figure S8(a), (b) and Fig. 4).

### Discovery of disease subtypes from multi-omics data sets

In this study, we combine three omics data sets (669 clinical variables, 4258 mRNA expressions and 438 miRNA expressions) from a cohort of 319 lung disease patients. To estimate the number of clusters, Additional file 1: Figure S9A-D presents the Gap statistics and the incremental differences in each omics data set for different comparisons. Almost all of the results clearly indicate 3 clear clusters in each omics data sources. Additional file 1: Figure S8 shows feature topology plots of the pairwise comparison of clustering results from each omics data set. The pie charts of the clusters describe the composition of existing diagnoses from clinicians. The three clusters identified from mRNA data and miRNA data are highly consistent with only 38 off-diagonal samples (38/319 = 12 %), while the comparison of mRNA vs clinical and miRNA vs clinical show heterogeneous clustering with 121 (38 %) and 150 (47 %) off-diagonal samples (Additional file 1: Figure S8). As a result, we merge mRNA and miRNA data for combined cluster analysis and compare with the clustering result from the clinical data in Fig. 4. The result shows three consensus clusters (cluster A, 76 samples mostly COPD; cluster I, 80 samples mostly ILD; cluster E, 43 samples of intermediate subtype) and six off-diagonal differentially defined clusters from the two omics data sources. Noticeably, 18 samples (cluster G) are determined ILD-like from clinical data but are viewed as COPD-like from mRNA + miRNA clustering. Similarly, 11 samples (cluster C) are viewed as COPD-like in clinical clustering but are ILD-like in mRNA + miRNA clustering. COPD and ILD are considered to have distinct disease mechanisms because of their extremely divergent phenotypic patterns despite similar risk factors as well as the presence of a combined emphysema and pulmonary fibrosis overlapping syndrome [20]. This makes it possible that omics measurements might help improve disease phenotyping. For example, the diagnoses of the first cluster (*n* = 121) by clinical data (sum of cluster A, B, and C) are mostly associated with COPD. With additional information from mRNA and miRNA expression, these 121 samples are further divided into clusters A, B and C. Overall, the result shows the limitation of current disease diagnosis that can be improved by large-scale clinical and transcriptomic measurements. The identified cluster E (and potentially also clusters B, D, F and H) may present a novel intermediate disease subtype with a disease mechanism different from existing COPD and ILD definitions.

A common issue in using omics data sets for disease subtype discovery is the reproducibility and potential presence of batch effects. To validate the finding, our analysis initially started with a first training cohort of 91 patients and was then validated in a second testing cohort of 228 patients. The proposed comprehensive validation scheme is comprised of the three phases of discovery, prediction and validation. A first training cohort produces clustering and MDS coordinates results, and thereby the MDS coordinates are directly applied to the testing cohort for clustering as the prediction phase. In the validation phase, clustering results are produced within the testing cohort. In our analysis, clustering results in the prediction and the validation phase show high reproducibility with a consistently identified intermediate disease subtype (see Additional file 1: Figure S6). Additional file 1: Figure S6(a) demonstrates a workflow of three phases of discovery, prediction and validation. Additional file 1: Figure S6(b) demonstrates the discovery phase of clustering results from the training cohort. The model is then applied to the testing cohort for clustering as the prediction phase in Additional file 1: Figure S6(c). Additional file 1: Figure S6(d) shows a validation phase of clustering result within the testing cohort. By comparing Additional file 1: Figure S6(c) and (d), the result shows high reproducibility with a consistently identified intermediate disease subtype. To analytically measure a level of concordance between clusters in Fig. 2c and d, we employ adjusted rand index (ARI) [21]. The estimated ARIs are 0.764 for the clinical data set and 0.43 for the three clusters in the transcriptomic data set (mRNA + miRNA). Taken together, we conclude that the discovered clusters from the training and testing cohort preserve common characters, which enable the combination of all the samples (*n* = 319) from the three batches in the pooled analysis, as shown in Fig. 4.

### Discovery of discriminant phenome features

We focus our analysis on the three consistent clusters, hereafter referred to as Cluster A, E and I. Table 1 shows the group means of 12 selected demographic and clinical variables for the three clusters and their pairwise as well as overall ANOVA p-values. Patients in cluster E were likely to be younger (average age 55 years) when compared to clusters A and I (65.7 and 66.1 years respectively) and there were more females (65.1 %) compared to A and I (39.5 % and 30 % respectively). In contrast, patients in cluster A had a more obstructive pattern in their pulmonary function test (average FEV1/FVC ratio 0.653) compared to those in clusters E and I (0.93 and 1.12 respectively). Similarly, quantitative CT analysis revealed that patients in cluster A were more likely to have emphysema (CT% emphysema 14.4 %) while those in cluster I were more likely to have high lung reticular volumes (662 mL). In this case, patients in cluster E seemed to express an intermediate phenotype although the gender and age distributions do no suggest cluster E as a pure intermediate phenotype between cluster A and I.

**Table 1:** Summary of significant features grouped in each cluster (cluster A, E, and I)^a^

	Total	Cluster A	Cluster E	Cluster I	*P*-value	Cluster	Cluster	Cluster
	(*n* = 199)	(*n* = 76)	(*n* = 43)	(*n* = 80)	ANOVA	A & E	A & I	E & I
Age, yrs	63.5	65.7	55	66.1	2.91E-07	7.68E–02	1.24E–07	5.05E–02
Gender, % female	41.2	39.5	65.1	30	7.51E–04	2.44E–02	7.23E–01	7.42E–04
Body Mass Index, BMI	28.6	28	27.5	29.8	3.29E–02	1.00E + 00	7.24E–02	1.07E–01
FEV1 % predicted	61.7	48	64.3	73.4	1.12E–13	4.37E–05	1.53E–13	3.61E–02
FVC % predicted	69.2	72.4	69.7	65.8	1.85E–02	1.00E + 00	1.79E–02	2.93E–01
FEV1/FVC ratio	0.9	0.653	0.93	1.12	3.62E–29	1.59E–09	1.49E–25	2.75E–09
DLCO	53.9	59.2	57.3	47	2.55E–03	1.00E + 00	8.14E–03	1.46E–02
Total lung capacity, mean	5.28	6.55	4.87	4.19	4.69E–19	1.16E–07	2.32E–18	4.98E–02
CT % emphysema	7.19	14.4	1.88	1.01	4.46E–13	1.73E–07	3.77E–11	3.70E–01
Lung reticular volume, ml	309	63.8	198	662	5.86E–17	2.22E–02	3.03E–16	2.32E–07
Diagnosis, % IPF	38.7	1.32	9.3	90	4.01E–33	1.16E–01	6.00E–28	6.75E–18
Diagnosis, % Emphysema	19.6	43.4	14	0	4.29E–11	3.20E–03	1.12E–10	1.99E–03

^a^The average values of 12 selected demographic and clinical variables in each sub–cluster groups, *p*–values from Kruskal–Wallis ANOVA (all three groups) and *p*-values from Kruskal-Wallis rank sum test (paired wise groups)

### Gene co-expression modules and pathway analysis demonstrates enrichment for inflammatory and immune related annotations in Cluster E

We performed one-way ANOVA analysis for each mRNA and miRNA feature based on cluster A (COPD), E (Intermediate) and I (ILD) labels, and identified 1684 statistically significant (p-value adjusted by Bonferroni  < 1e-10) features in the gene expression (*n* = 1596) and miRNA (*n* = 88). We further performed gene co-expression cluster analysis using partition around medoids (PAM) to identify four gene and miRNA modules in Fig. 5 (gap statistics analysis in Additional file 1: Figure S10 clustered into four gene modules). Note that we intentionally turned the 88 miRNAs to opposite direction (by multiplying the expression intensities by -1) in the cluster analysis to account for the fact that most miRNAs have inhibitory effects on mRNA expression. As a result, expressions of miRNAs in a module [5] have a clear negative correlation with the remaining mRNAs (blue) in Fig. 5.

In order to look more deeply into the biological pathways differentiating the microarray samples in cluster E from those in cluster I (Fig. 5) we focused on the genes in module two, since this was the most distinctive of all studied modules. By using Ingenuity Pathway Analysis (IPA, see Methods for more detail), we identified enrichment for a large number of immune related annotations in module two genes when comparing samples in cluster E relative to those in cluster I. These annotations were largely related to immune cell trafficking, predominantly leukocyte activation, migration, movement and chemotaxis as well as cell movement of phagocytes, neutrophils and myeloid cells (Additional file 1: Table S18). The large majority of these annotations showed an increase in their activation score (z-score), predicting the increase activation of these functions in cluster E subjects.

Given the enrichment for immune related annotations, we looked for potential drugs targeting overexpressed genes in module two and identified drugs with immunomodulatory and immunosuppressive effects (Additional file 1: Figure S12), medications that are currently not recommended for the treatment of IPF patients, the predominant group of patients in cluster E. Some of these drugs included hydroxichloroquine, a drug that has been largely used to treat rheumatoid arthritis and systemic lupus erythematous [22, 23]. Rituximab, a humanized monoclonal antibody which targets B cell and is commonly used to treat autoimmune disorders and Efalizumab, a formerly available immunosuppressant that was used to treat autoimmune disorders by inhibiting lymphocyte activation and cell migration. Taken together, these results demonstrate how iPF allowed us to identify a subset of patients with a predominant immune related phenotype that could potentially respond to immunomodulatory therapy.

### Simulation study to show advantages of iPF

To demonstrate that iPF is robust and accurate in disease subtype discovery using omics data, we simulated data sets with various degrees of variances and proportion of noise features, using the “clusterGeneration” package. Additional file 1: Figure S11 shows that feature fusion methods used in iPF (FF, FFspK and FFmClust) outperform traditional clustering methods. More details are shown in Additional file 1: Text S3.

## Discussion

In this paper, we have proposed a framework to integrate multi-omics data sources for disease subtype identification. Compared to existing methods (such as iCluster and Bayesian consensus clustering), iPF allows different variable types (binary, continuous, ordinal and multi-class), which are common in clinical variables. The method is also model-free, manages missing values without imputation techniques (i.e., pair-wise feature correlations can be estimated by excluding only samples with missing values), robust to data noises and provides effective visualization tools. Compared to the latent variable estimation in iCluster and posterior distribution simulation in Bayesian consensus clustering, the computational load of iPF is also much more affordable. Our hierarchical integrative strategy (Fig. 3) further allows analysis of homogeneous and heterogeneous clustering structure across multi-omics data.

We tested the iPF framework using two large multi-omic data sets obtained from lung tissues from well characterized patients with chronic lung diseases. One of the most relevant findings by iPF is the replication of the two most distinct disease subphenotypes in pulmonary medicine: COPD and ILD, diseases that represent the opposite spectrum of lung physiologic patterns, airflow obstruction in COPD and lung restriction in ILD. Currently, the diagnosis of obstructive and restrictive lung diseases relies mostly on clinical findings, pulmonary function test, radiologic studies and lung biopsies in selected cases [24]. Results from genomic analyses of lung tissues have not been included in diagnostic algorithms in chronic lung diseases mostly because of the added cost of these technologies, the difficulties to access lung tissue samples and the concept that an accurate diagnosis can be achieved by using the current diagnostic armamentarium in clinical practice and most commonly without the need to obtain a biopsy. Our findings demonstrate that iPF is a valuable tool not only because it was able to distinguish the known disease categories, but also because it allowed us to identify a previously unknown disease subphenotype of patients that clustered in between patients with COPD and ILD (see Additional file 1: Figure S19), characterized by the overexpression of genes predominantly associated with immune cell activation and trafficking. Interestingly, a large number of patients in this cluster had the clinical diagnosis of Idiopathic Pulmonary Fibrosis (IPF).

The identification of genes associated with immune cell activation in a subset of patients with IPF is relevant for various reasons. IPF is a fibrotic lung disease of unknown etiology, associated with high mortality rates [25]. IPF is thought to be caused by repeated cycles of alveolar epithelial cell injury followed by fibroblast recruitment, proliferation and extracellular matrix deposition [26]. In the past, IPF was considered to be a chronic inflammatory process, in part, due to the partial response observed with the use of immunosuppressive therapy in a small subset of patients, evidence that was limited to case reports [27]. Subsequently, a large randomized, controlled clinical trial demonstrated that immunosuppressive drugs actually increased the mortality and number of hospitalizations of IPF patients [28] and the use of these drugs was abandoned from clinical practice. Our pathway analysis revealed enrichment for immune related annotations when comparing samples between intermediate (cluster E) and ILD (cluster I) clusters (Fig. 4). These annotations were largely associated with immune cell trafficking, leukocyte activation, migration, movement and chemotaxis (For details, see Additional file 1: Table S18). The annotations mostly present high activation scores suggesting increased activation of these pathways in cluster E patients. The fact that some of the overexpressed genes in the intermediate cluster of ILD patients are targeted by immonompodulatory drugs such as hydroxichloroquine, Rituximab, and Efalizumab, suggest that a small subset of ILD patients, even among those with idiopathic pulmonary fibrosis (IPF), may actually benefit from immunomodulation. In this regard, iPF facilitates sub-categorizing patients on the basis of multi-level data sources beyond the conventional diagnostic tools, and identifying biologically associated functional annotations and new drugs effective to the subset of patients, namely, implementing personalized diagnosis and treatment of chronic lung diseases.

Integrative Phenotyping Framework has a few potential limitations. Firstly, the method applies dimension reduction and smooth techniques. The performance is expected to deteriorate if the omics throughput is too low (e.g. data from small number of clinical variables or a small assay). Secondly, several procedures in iPF are not fully automated and need expert decision. For example, the decision of the number of clusters is not always easy and is an intrinsic methodological difficulty for almost all clustering tools in real practice. In our lung disease application, clusterings from mRNA and miRNA were found to be almost identical and it was an easy decision to merge the two omics data sets in the hierarchical integration. In other applications, we expect that deciding on the homogeneous or heterogeneous clustering results may not always be clear-cut. Thirdly, the hierarchical integration is performed pairwisely, merging and comparing two at a time. The number of clusters may increase exponentially if many omics data sets are combined and all omics data generate heterogeneous clustering. However, such complexity may be an intrinsic biological fact and cannot easily be accommodated by statistical models. Finally, our findings in the first multi-omics data set were only validated “in-silico” in the second multi-omics data set. While it would have been ideal to add a second validation method using an alternative gene expression platform (i.e. reverse transcriptase, polymerase chain reaction – qRT-PCR), we felt that the strength of our results made unnecessary the addition of an alternative validation method.

The integration of multi-omics data sources using the iPF framework has immense potential to advance the field of personalized medicine by confirming clinical diagnoses, aid in the identification of new disease subphentoypes, provide biological insights, and new targets for drug therapy. However, despite the impressive reproducibility of our findings across two large data sets, additional studies focusing on validating our results in other large data sets will be required before the iPF framework can be applied to daily clinical practice.

## Methods

### Integrative phenotyping framework (iPF)

#### Data pre-processing and feature integration

The overall workflow and diagram are demonstrated in Figs. 1 and 2. For pre-processing, each omics data set was normalized separately. Non-expressed (low mean intensities) and non-informative (low standard deviations) features were filtered. We consider the integration of *M* different types of high-throughput omics data sets, denoting these by ${X}^{(m)}\kern0.5em =\kern0.5em {\left\{{x}_{ij}^{(m)}\right\}}_{\left|{I}_m\left|\kern0.5em \times \kern0.5em \right|J\right|}$ data set of the *m*^th^ omics, where *x*_*ij*_^(*m*)^ is the intensity of feature *i* and sample *j* for *i* ∈ *I*_*m*_, *j* ∈ *J*, and *m* = 1, 2, … , *M*. Denote by ${\overset{\rightharpoonup }{X}}_i^{(m)}\kern0.5em =\kern0.5em \left\{{x}_{ij}^{(m)},\kern0.5em \dots \kern0.5em ,{x}_{i\left|J\right|}^{(m)}\right\}$ the feature vector of the *i*^*th*^ feature in the *m*^*th*^ omics data set. The omics data are vertically concatenated such that *X* = {*x*_*ij*_}_|*I*| × |*J*|_ is the combined data set of all *M* data sets, where *I* = ∪ _*m* = 1_^*M*^*I*_*m*_. (Fig. 2a). All features are standardized to have zero mean and unit variance to avoid scaling issues or dominance of certain omics types.

#### MDS dimension reduction and feature smoothing

Based upon the combined data set *X*, we calculate the feature dissimilarity (distance) matrix by

$$ D\kern0.5em =\kern0.5em \frac{\left({\left\{1\right\}}_{\left|I\left|\times \right|I\right|}-R\right)}{2}\kern0.5em =\kern0.5em {\left\{{d}_{i{i}^{\prime }}\right\}}_{\left|I\left|\times \right|I\right|}, $$

where *R* is the correlation matrix (defined in Additional file 1: Table S14) between different variable types such as continuous, ordinal, binary and multi-class categorical (Fig. 2b). Multi-dimensional scaling (non-metric MDS) is then applied to project all concatenated features onto a two-dimensional space:

${G}_X\kern0.5em \left({\overset{\rightharpoonup }{X}}_i^{(m)}\right)\kern0.5em =\kern0.5em \left({u}_{i1}^{(m)},\kern0.5em {u}_{i2}^{(m)}\right),\kern0.5em where\kern0.5em {G}_X\kern0.5em :\kern1em {R}^J\kern0.5em \to \kern0.5em {R}^2\kern1em ,\kern1em i\kern0.5em \in \kern0.5em {I}_m\kern0.5em \mathrm{and}\kern0.5em m\kern0.5em =\kern0.5em 1,\kern0.5em \dots \kern0.5em ,M.$ Note that the MDS mapping is performed based on the combined data set *X*. MDS coordinates after feature projects are comparable across different omics data sets (Fig. 2c), an important property to allow clustering comparison across omics data sets in a later step. Feature intensities are represented by gradient color: red, yellow and blue represent high, intermediate and low intensities respectively. After dimension reduction, we apply a nonparametric smoothing technique to intensities at MDS coordinates using generalized additive model (GAM) [29] and create a Feature Topology Plot (FTP) using the smoothed intensities in 2D space (See Additional file 1: Figure S5(b)). The smoothing method applies a thin plate spline penalty as the basis (TPRS) [30]. It averages response values in a neighborhood and plays an essential role for iPF to be robust against randomly noisy features.

This workflow can be seen as converting integrated feature vectors into a smoothed 2D image in the MDS space, and includes multiple benefits. First, highly correlated features are encouraged to concentrate in a small region in the 2D image. So when we measure the distance (correlation) between two subjects based on 2D space, these highly correlated feature will contribute less than they do at the original feature space. It is challenging to characterize high-dimensional covariance among thousands or tens of thousands features. Instead we project feature vectors into the 2D space (MDS), which help to attenuate effects of highly correlated features. Second, the smoothing technique reduces noise effects and thus enhances the efficiency of clustering analysis. Additional file 1: Text S3 presents various numerical simulations for sensitivity and robustness analyses of the proposed Feature Fusion technique (= Feature concatenation + Dimension reduction + Feature smoothing). Details of smoothing and generation of Feature Topology Plots are available in Additional file 1: Text S2.

#### Cluster analysis for subtype discovery and visualization

In Fig. 2 (d), we can generate the smoothed intensities of each patient *j* in the *m*^th^ omics data set. Suppose we scale the MDS coordinates to the unit square (x, y coordinates between 0 and 1). We represent the smoothed intensity vector (of length (n + 1)^2^) of patient *j* in the *m*^th^ omics data set as ${c}_j^{(m)}\kern0.5em =\kern0.5em \left\{{\widehat{f}}_j^{(m)}\kern0.5em \left({\scriptscriptstyle \frac{s}{n}},\kern0.5em {\scriptscriptstyle \frac{t}{n}}\right)\right\},$ where ${\widehat{f}}_j^{(m)}\kern0.5em \left({\scriptscriptstyle \frac{s}{n}},\kern0.5em {\scriptscriptstyle \frac{t}{n}}\right)$ is the smoothed intensity at the $\left({\scriptscriptstyle \frac{s}{n}},\kern0.5em {\scriptscriptstyle \frac{t}{n}}\right)$ MDS coordinate (*s* = 0,1,…, *n* and *t* = 0,1,…,*n*). To perform cluster analysis to assign patients into clusters based on the *m*^th^ omics data set, we adopt the dissimilarity measure between any two patients *i* and *j* as *D*_*j*,*j* '_^(*m*)^ = 1 − *cor* (*c*_*j*_^(*m*)^, *c*_*j* '_^(*m*)^), where *cor*(⋅,⋅) is the Pearson correlation of two vectors. Partition around medoids (PAM) [31] is then applied to cluster patients into potential disease subtypes. The number of clusters is determined by Gap statistics [32] for each omics data set. Figure 2e (Step vii to viii) shows a schematic demonstration that each of the two omics and clinical data sets generates three clusters of patients. One can further average the Feature Topology Plots in each cluster to visually show the cluster patterns (Step ix in Fig. 2e). For example, Additional file 1: Figure S8(a) shows almost identical cluster pattern visualization from mRNA and miRNA expression data sets, which are further validated by the confusion matrix with few off-diagonal patients. This justifies combining mRNA and miRNA for clustering and comparison with clustering from the clinical data in Fig. 4.

#### Integrative strategy for multi-omics clustering

One major difficulty of disease subtype discovery using multi-omics data is the possible heterogeneity of clustering results from different omics data. The above iPF framework and visualization can handle integration of *M* = 2 omics data sets. When *M* ≥ 3, we propose to first compare all possible pairs of omics data sets (Fig. 3 Step 1). In our motivating example, three pairwise comparisons are demonstrated in Additional file 1: Figure S8. It is clear that mRNA and miRNA gives almost the same clustering results while clustering from clinical data is very different from mRNA and miRNA. As a result, we combine mRNA and miRNA (Fig. 3 Step 2) and compare with clinical data (Fig. 3 Step 3) to generate the final result shown in Fig. 4.

### Biomarker detection and functional annotation

After patient clusters are determined from mRNA + miRNA and clinical data clustering in Fig. 4, we select the unambiguous clusters (cluster A: COPD; cluster I: ILD; cluster E: novel intermediate subtype) for further biomarker detection and functional annotation. One-way analysis of variance [33] is applied to detect significant biomarkers associated with the three clusters (p-value adjusted by Bonferroni < 1e-10). The detected biomarkers are clustered using partition around medoids (PAM) to identify co-expressed gene modules and the number of gene modules is determined by Gap statistics (Additional file 1: Figure S10). Functional annotation is performed using Ingenuity Pathway Analysis (IPA) software.

### Methods for pathway enrichment analysis in cluster E

In order to identify underlying biological pathways distinguishing the microarray samples from patients in cluster E relative to cluster I, we performed IPA and the Ingenuity Downstream Effects Analysis. The downstream effects analysis is based on prior knowledge of expected causal effects between genes and biological functions stored in the Ingenuity® Knowledge Base. This analysis examined genes in the selected modules that are known to affect biological functions and compared their direction of change (i.e. expression in cluster E samples relative to cluster I) to that expected from the literature. In this analysis, if the observed direction of change is mostly consistent with a particular activation state of a biological function (“increased” or “decreased”), then a prediction is made about that activation state. For each biological function, an activation z-score is computed. The activation z-score is used to infer likely activation states of biological functions based on comparison with a model that assigns random regulation directions.

## Conlusion

We present an integrative analysis tool to inter-connect disease subphenotypes and visualize feature intensity patterns. Using a large dataset of lung samples with parallel genomic and phenomic data, we show this integrative phenotyping framework (iPF) can lead to successful feature discovery and integrative clustering in high dimensional space. Applying iPF to large data sets, we identify a subphenotype of patients with Chronic Obstructive Pulmonary Disease and Interstitial Lung Disease, characterized by overexpression of genes associated with inflammatory and immune responses.

## Additional files

Additional file 1:
**Text S1.** Materials and data collection. **Text S2.** Details of smoothing and Feature Topology Plots (FTP). **Text S3.** Simulation setting to evaluate iPF. **Text S4.** Comprehensive validation scheme for iPF. **Figure S5.** (A) An illustration of integrated omics data sets, (B) A workflow to generate future topology plot (FTP). **Figure S6.** Flowchart of validation scheme for Integrative phenotyping framework for multiple omics data sets. **Figure S7.** An example of iPF that utilizes fused multiple data sets at the stage (vi). **Figure S8.** Examples of iPF using various combinations of the omics data sets (pooled analysis). **Figure S9A.** The gap statistics and its scree plot to choose the optimal number of clustering (clinical and miRNA data). **Figure S9B.** The gap statistics and its scree plot to choose the optimal number of clustering (mRNA and miRNA data). **Figure S9C.** The gap statistics and its scree plot to choose the optimal number of clustering (mRNA and clincal data). **Figure S9D.** The gap statistics and its scree plot to choose the optimal number of clustering (clincal data and combined data of mRNA and miRNA). **Figure S10.** The best choice of the number of feature modules. **Figure S11.** Simulation study shows robust true feature discovery in “Feature Fusion”. The x-axis represents multiplication levels of noise features. The y-axis represents average ARIs from 100 simulations. Each figure is generated based on simulation scenarios of the different number of true features (e.g., 200, 400, and 600, respectively). **Figure S12.** Immunomodulating drugs target overexpressed genes in module two. **Table S13.** The description of mRNA and miRNA lung disease data. **Table S14.** Various correlation types depending on variable attributes. **Table S15.** The demographic summary of clinical features in each sub-cluster. **Table S16.** Target gene enrichment analysis (via Fisher exact test) related to twelve. **Table S17.** Regression analysis on target miRNA features, and coefficient of determination significant miRNA features. **Table S18.** The top disease or functional annotations associated with genes in module two in Cluster E patients. **Figure S19.** Basic consensus clustering using only gene expression data. (DOCX 6398 kb) Additional file 2:
**This file includes GSM IDs of GSE47460 that were collected to analyze in this paper.** (XLSX 43 kb) Additional file 3:
**This file includes 669 clinical variables analyzed in this paper.** (XLSX 45 kb) Additional file 4:
**This is the first survey form to screen patients. Each colored label on the right hand side corresponds to variable names that appear in the clinical data set.** (PDF 9214 kb) Additional file 5:
**This is the second survey form to screen patients. Each colored label on the right hand side corresponds to variable names that appear in the clinical data set.** (PDF 774 kb)

## Abbreviations

iPF: Integrative phenotying framework
IPF: Idiopathic Pulmonary Fibrosis
COPD: Chronic obstructive lung disease
ILD: Interstitial lung disease
NSIP: Non Specific Interstitial Pneumonia
HP: Hypersensitivity Pneumonitis
COP: Cryptogenic Organizing Pneumonia
RB-ILD: Respiratory Bronchiolotis-associated Interstitial Lung Disease
CVD-ILD: Collagen Vascular Disease—associated Interstitial Lung Disease
DIP: Desquamative Interstitial Pneumonia
AIP: Acute Interstitial Pneumonia
TCGA: The Cancer Genome Atlas
MDS: Multi-dimensional scaling.
