# Supplementary material for: Integrative phenotyping framework (iPF): integrative clustering of multiple omics data identifies novel lung disease subphenotypes
Source: BMC Genomics. 2015 Nov 11;16:924. doi: 10.1186/s12864-015-2170-4 (PMC4642618; doi:10.1186/s12864-015-2170-4)
Supplement: Additional file 1: — Text S1. Materials and data collection. Text S2. Details of smoothing and Feature Topology Plots (FTP). Text S3. Simulation setting to evaluate iPF. Text S4. Comprehensive validation scheme for iPF. Figure S5. (A) An illustration of integrated omics data sets, (B) A workflow to generate future topology plot (FTP). Figure S6. Flowchart of validation scheme for Integrative phenotyping framework for multiple omics data sets. Figure S7. An example of iPF that utilizes fused multiple data sets at the stage (vi). Figure S8. Examples of iPF using various combinations of the omics data sets (pooled analysis). Figure S9A. The gap statistics and its scree plot to choose the optimal number of clustering (clinical and miRNA data). Figure S9B. The gap statistics and its scree plot to choose the optimal number of clustering (mRNA and miRNA data). Figure S9C. The gap statistics and its scree plot to choose the optimal number of clustering (mRNA and clincal data). Figure S9D. The gap statistics and its scree plot to choose the optimal number of clustering (clincal data and combined data of mRNA and miRNA). Figure S10. The best choice of the number of feature modules. Figure S11. Simulation study shows robust true feature discovery in “Feature Fusion”. The x-axis represents multiplication levels of noise features. The y-axis represents average ARIs from 100 simulations. Each figure is generated based on simulation scenarios of the different number of true features (e.g., 200, 400, and 600, respectively). Figure S12. Immunomodulating drugs target overexpressed genes in module two. Table S13. The description of mRNA and miRNA lung disease data. Table S14. Various correlation types depending on variable attributes. Table S15. The demographic summary of clinical features in each sub-cluster. Table S16. Target gene enrichment analysis (via Fisher exact test) related to twelve. Table S17. Regression analysis on target miRNA features, and coefficient of determination significant miRNA feat [file 12864_2015_2170_MOESM1_ESM.docx]

**Supporting Information**

**Text S1: Materials and data collection**

We acquired flash frozen lung tissues, computed tomography (CT) data, and clinical data on 319 samples from the Lung Genomics Research Consortium (LGRC, https://www.lung-genomics.org/). These samples were initially given major clinical diagnoses of either interstitial lung disease (ILD) or chronic obstructive pulmonary disease (COPD) based on their clinical, pathologic, and radiographic data (136 COPD and 183 ILD). 669 clinical variables were collected by questionnaires (demographic, medical history, family history, smoking history, concomitant therapy, symptom, SF-12 health, St. Georges respiratory, environmental, and occupational), tests (six-minute walk test, cardiopulmonary exercise test, PFT, blood test, and CT scan), and diagnosis reports (central and local pathology, clinical report). More details on the data collection are publicly available (http://www.ltrcpublic.com/clinical_IQ.htm; SI_survey_form_1.pdf” and “SI_survey_form_2.pdf). Gene expression and miRNA data sets were generated through Agilent microarray platforms (for details, refer to S13 Table; GSE47460 and GSE72967) After we matched probes with gene symbols and applied loess normalization [[1](#_ENREF_1)] to two Agilent platforms, we obtained 15,966 genes and 937 miRNAs. For gene expression, redundant (e.g. non-expressed and/or non-informative) gene expression features with mean (< 7) and standard deviation (< 0.4) were removed. For miRNAs, we filtered the miRNAs probes based on the mean expression (< 1.7) across a total of n samples. We analyzed 669 clinical variables, 4,258 gene expressions and 438 miRNA genes from 319 samples.

**Text S2: Details of smoothing and Feature Topology Plots (FTP)**

Let $X_{j}^{\left( m \right)}=(X_{1j}^{\left( m \right)},\ldots,X_{\left| I_{m} \right|j}^{\left( m \right)})$ denote an intensity vector for sample $j$ and the *m*^th^ omics data, where $i\in I_{m}, j\in J, m=1,..., M$ and $\left| I_{m} \right|$ is the size of feature set $I_{m}$ for the *m*^th^ omics data. Denote by $\left( u_{i1}^{(m)},u_{i2}^{(m)} \right)$ the two-dimensional MDS coordinates of feature $i\in I_{m}$ in the *m*^th^ omics data. With fixed sample *j* and omics data *m*, we fit a generalized additive model using a thin plate spline penalty on $X_{j}^{\left( m \right)}$ and $\left( u_{i1}^{(m)},u_{i2}^{(m)} \right)$:

$E(X_{ij}^{\left( m \right)}|u_{i1}^{\left( m \right)},u_{i2}^{(m)})=\beta_{j0}^{(m)}+ \beta_{j1}^{(m)}s_{j1}^{(m)}\left( u_{i1}^{\left( m \right)},u_{i2}^{(m)} \right)+ \beta_{j2}^{(m)}s_{j2}^{(m)}\left( u_{i1}^{\left( m \right)},u_{i2}^{(m)} \right)+ \cdots+\beta_{jp_{j}}^{(m)}s_{jp_{j}}^{(m)}\left( u_{i1}^{\left( m \right)},u_{i2}^{(m)} \right)$,

where $i\in I_{m}$ and $p_{j}$ is the optimal number of spline bases. We applied penalized thin plate regression splines for the generalized additive models using the “mgcv” package in R [[8](#_ENREF_8)]. The estimated coefficients and splines derived from the penalized approach are used to smooth the intensity estimates:

$$\hat{f}_{j}^{(m)}\left( x_{1},x_{2} \right)=\hat{\beta}_{j0}^{(m)}+ \hat{\beta}_{j1}^{(m)}\hat{s}_{j1}^{(m)}\left( x_{1},x_{2} \right)+ \hat{\beta}_{j2}^{(m)}\hat{s}_{j2}^{(m)}\left( x_{1},x_{2} \right)+ \cdots+\hat{\beta}_{jp_{j}}^{(m)}\hat{s}_{jp_{j}}^{(m)}\left( x_{1},x_{2} \right)$$

for $i\in I_{m}, j\in J, m=1,..., M, \mathrm{and} \left( x_{1},x_{2} \right)\in R^{2}$. In the model, features with missing values are allowed. The top panel of Supplementary Figure 1b illustrates feature intensities at scaled MDS coordinates $U^{'(m)}$, adjusted by the minimum and maximum of MDS coordinates of $u_{i1}^{(m)}$ and $u_{i2}^{(m)}$, respectively, where$U^{'(m)}=\left\{ u_{ik}^{'(m)} \right\}_{\left| I_{m} \right|\times2}$ and $u_{ik}^{'(m)}=\left\{ \begin{aligned} u_{i1}^{'(m)}=\frac{u_{i1}^{(m)}-min\left( u_{i1}^{(m)} \right)}{\max\left( u_{i1}^{(m)} \right)-min\left( u_{i1}^{(m)} \right)} \\ u_{i2}^{'(m)}=\frac{\left( u_{i2}^{(m)} \right)-min\left( u_{i2}^{(m)} \right)}{\max\left( u_{i2}^{(m)} \right)-min\left( u_{i2}^{(m)} \right)} \end{aligned} \right.$ for $i\in I_{m}, m=1,..., M$, and $k=1 \mathrm{and} 2$. The feature intensities in the middle panel in Supplementary Figure 1b represent predicted smoothed intensities, $h_{j}^{(m)}=\left\{ \hat{f}_{j}^{(m)}\left( \frac{s}{n},\frac{t}{n} \right) \right\}_{(n+1)\times(n+1)}$ at lattice points in $G=\left\{ \left( \frac{s}{n},\frac{t}{n} \right) \right\}_{(n+1)\times(n+1)}$ for $s=0,\ldots,n, t=0,\ldots,n, i\in I_{m}, j\in J, m=1,..., M, \mathrm{and}$ $n$ refers to the grid number (we use default=19). To make sure that lattice points in $G$ account for a distribution of MDS coordinates in $U^{'(m)}$, we remove some grid points in $G$, whose distances from any points in $U^{'(m)}$ are greater than 0.2. As a result, the smoothed intensity plot does not cover entire two-dimensional space (i.e., The region remains white) as shown in the middle and bottom figure of Supplement Figure 1b.

**Text S3: Simulation setting to evaluate iPF**

We adopted a simulation scheme introduced by Qiu et al. [[2](#_ENREF_2)] to evaluate iPF. Simulated data sets were generated by the “clusterGeneration” package in R (http://www.r-project.org) with an adjustment to degrees of noise features to mimic complexity of real data sets. A total of M=5 different omics data sets that hold $\left| J \right|$=100 samples belonging to five disease subtypes (20 samples each) were simulated. In each simulated data set, numNonNoisy=200, 400, 600, features from five pre-defined gene modules were simulated which lead to five disease subtypes. Extra noise features (numNoisy) (e.g. numNoisy is in proportion to numNonNoisy, numNoisy = 200 (=numNonNoisy x 1), 400 (=numNonNoisy x 2), 600 (=numNonNoisy x 3) and 800 (=numNonNoisy x 4), if numNonNoisy = 200) were sampled from uniform distribution. The parameter “sepVal” that determines the degree of separation of five disease subtypes was set at 0.7.

To evaluate clustering accuracy of iPF, we integrated the five simulated data sets and applied eight different clustering methods (“naïve”, “spK”, “mClust” “var”, “pca”, “FF”, “FFspK” and “FFmClust”) to cluster samples into five subtypes and compare to true clustering labels. K-means clustering (“naïve”) [[3](#_ENREF_3)], sparse K-means clustering (“spK”) [[4](#_ENREF_4)] and model-based clustering (“mClust”) [[5](#_ENREF_5)] are applied to all original features of integrated five omics data sets (i.e. clustering based on $X_{j}=(X_{j}^{\left( 1 \right)},\ldots, X_{j}^{\left( 5 \right)})$ for each sample, *j*). The method labeled with “var” means we sort features by variance in decreasing order and in turn apply K-means clustering to only the top 100 features in each omics data set. The method of “pca” means we perform principal component analysis (PCA) to select the top three PCs to which we apply K-means clustering. The methods (“FF”, “FFspK” and “FFmClust”) indicate we apply Kmeans, sparse-Kmeans and model-based clustering to smoothed feature intensities (Feature Fusion). More specifically, smoothed intensities on the grid points are $h_{j}^{(m)}=\left\{ \hat{f}_{j}^{(m)}\left( \frac{s}{n},\frac{t}{n} \right) \right\}_{(n+1)\times(n+1)}$ as in Supplementary material B. To numerically assess, we calculated an adjusted Rand index (ARI) that measures the similarity between inferred clustering labels and underlying true subtype labels. The simulations were repeated 100 times, and average ARIs are presented in Supplementary Figure 7. Supplementary Figure 7 shows the three methods related to Feature Fusion methods (“FF”, “FFspK” and “FFmClust”) clearly better perform compared to the other methods. Especially the Feature Fusion methods are fairly robust to effects of noise features, even if the number of noisy feature increases. Therefore, the Feature Fusion technique promotes effective integrative clustering, which is mostly attributed to dimension reduction and smoothing.

**Text S4: Comprehensive validation scheme for iPF**

As in Supplementary Figure 2a-d, we first divided all samples (n=319) into two groups: training set (n=91, Batch 1) and testing set (n=228, Batch 2). In the discovery phase, we applied iPF to the training data, and identified three distinct patterns of feature topology plot (FTP) in clinical and transcriptome (mRNA +miRNA) data sets. In the prediction phase, we produce FTPs of each testing sample (n=228) that utilize the MDS coordinates derived from training data sets. To validate if training set and testing set share homogenous variation structures in 2D space, we compared FTPs and disease proportions in pie charts of both training and testing set. In Supplementary Figure 2b and 2c, we found visual patterns of FTPs of both the training and testing set look alike, and the similar disease compositions across the nine sub-clusters were observed. After the visual confirmation, we independently applied iPF to the testing set. We thus estimated new feature relocations (MDS coordinates) of the testing data, and thereby created de novo FTPs of the testing set. These de novo FTPs are, therefore, no longer associated with the FTPs derived from the training set. Interestingly, both FTPs in Supplementary Figure 3b and 2d appear similar. This result implies both training and testing data set are formed in the homogenous distance structure of whole features. We measured concordance levels of the sub-clusters that are generated in the prediction phase and the validation phase by means of the adjusted rand index (ARI). Using 228 samples of the testing data set, we obtained ARI=0.76 for three clusters of clinical data set, and ARI=0.43 for three clusters of transcriptome data set. Taken together, we conclude that the sub-clusters of the training and testing data set adequately represents the common patterns of clustering, which resultingly provides a rationale to perform pooled analysis, such that we applied iPF to all patients of both two batches in Figure 4.


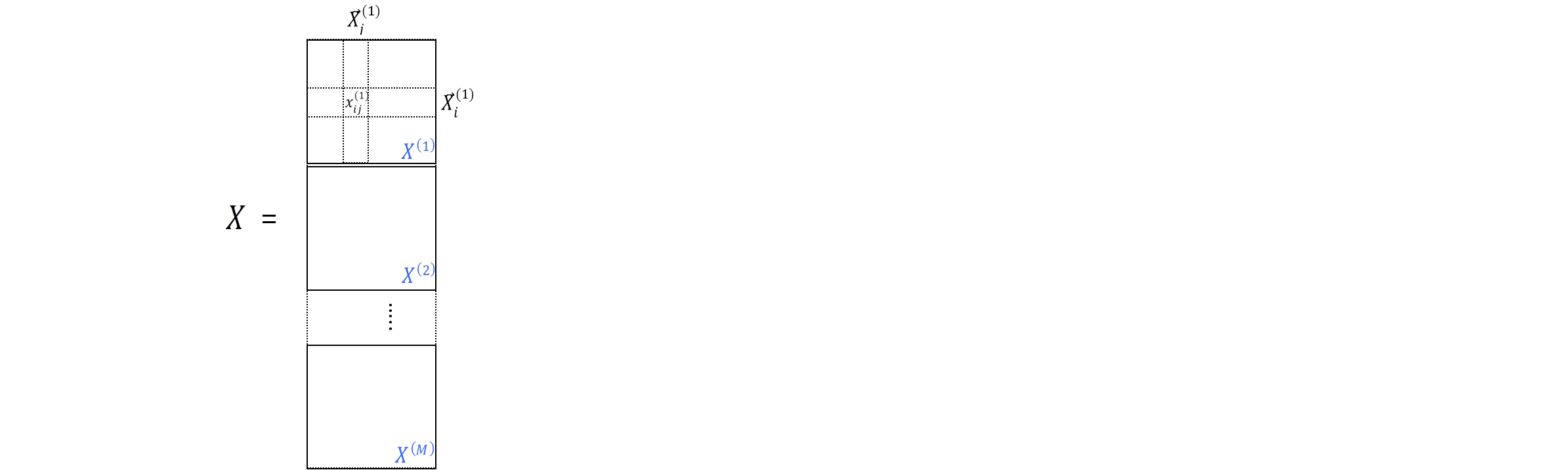

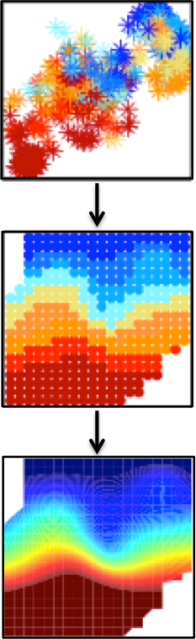


a.

b.

**Figure S5:** (a) An illustration of integrated omics data sets
(b) A workflow to generate feature topology plot (FTP)


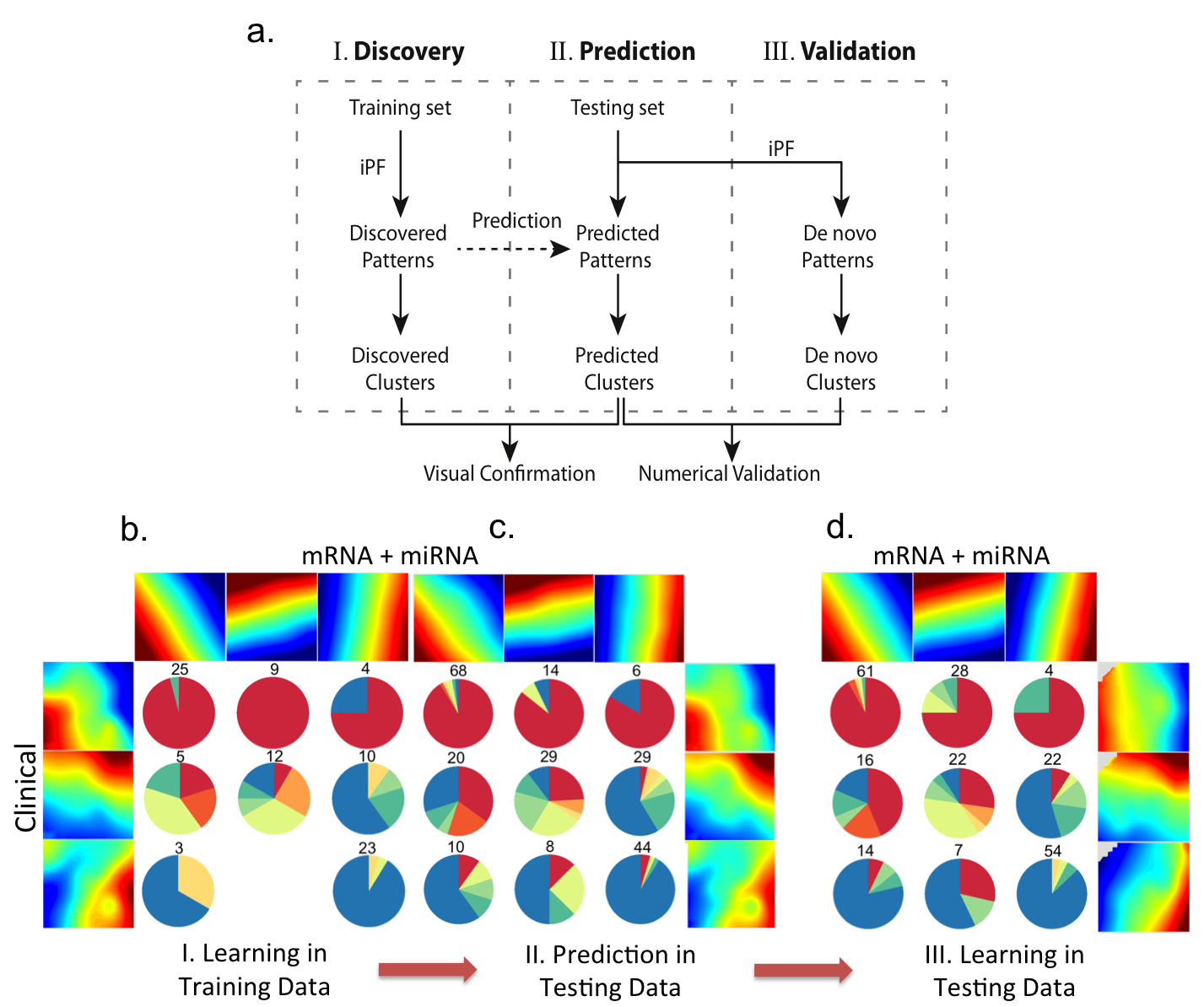


**Figure S6:** Flowchart of validation scheme for Integrative phenotyping framework for multiple omics data sets

(Clinical, Gene expression + miRNA)

**Figure S7:** An example of iPF that utilizes fused multiple data sets at the stage (vi)


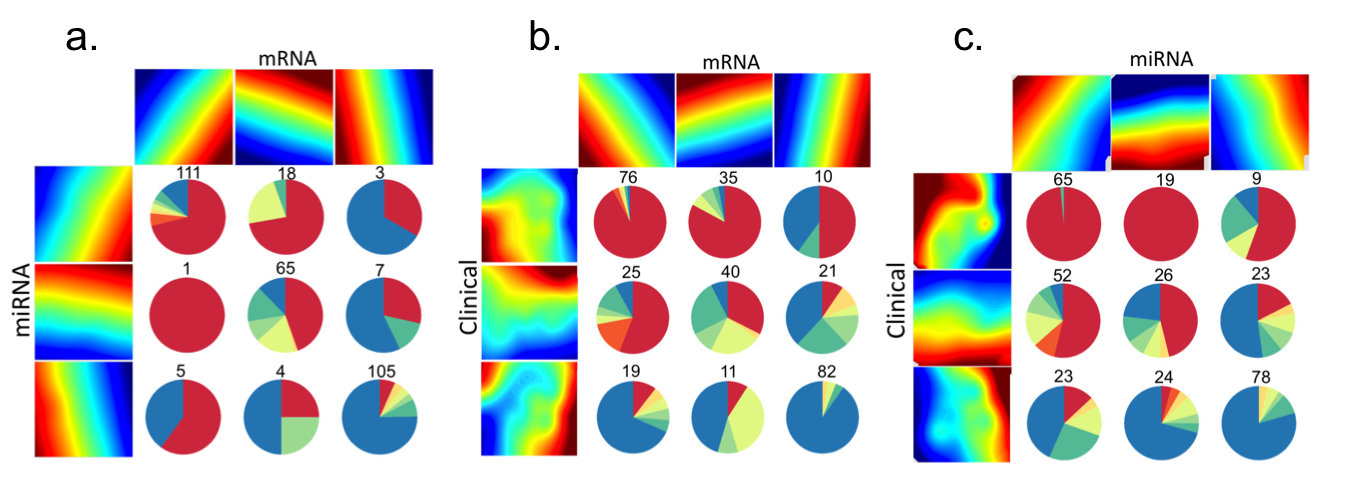


**Figure S8:** Examples of iPF using various combinations of the omics data sets (pooled analysis)


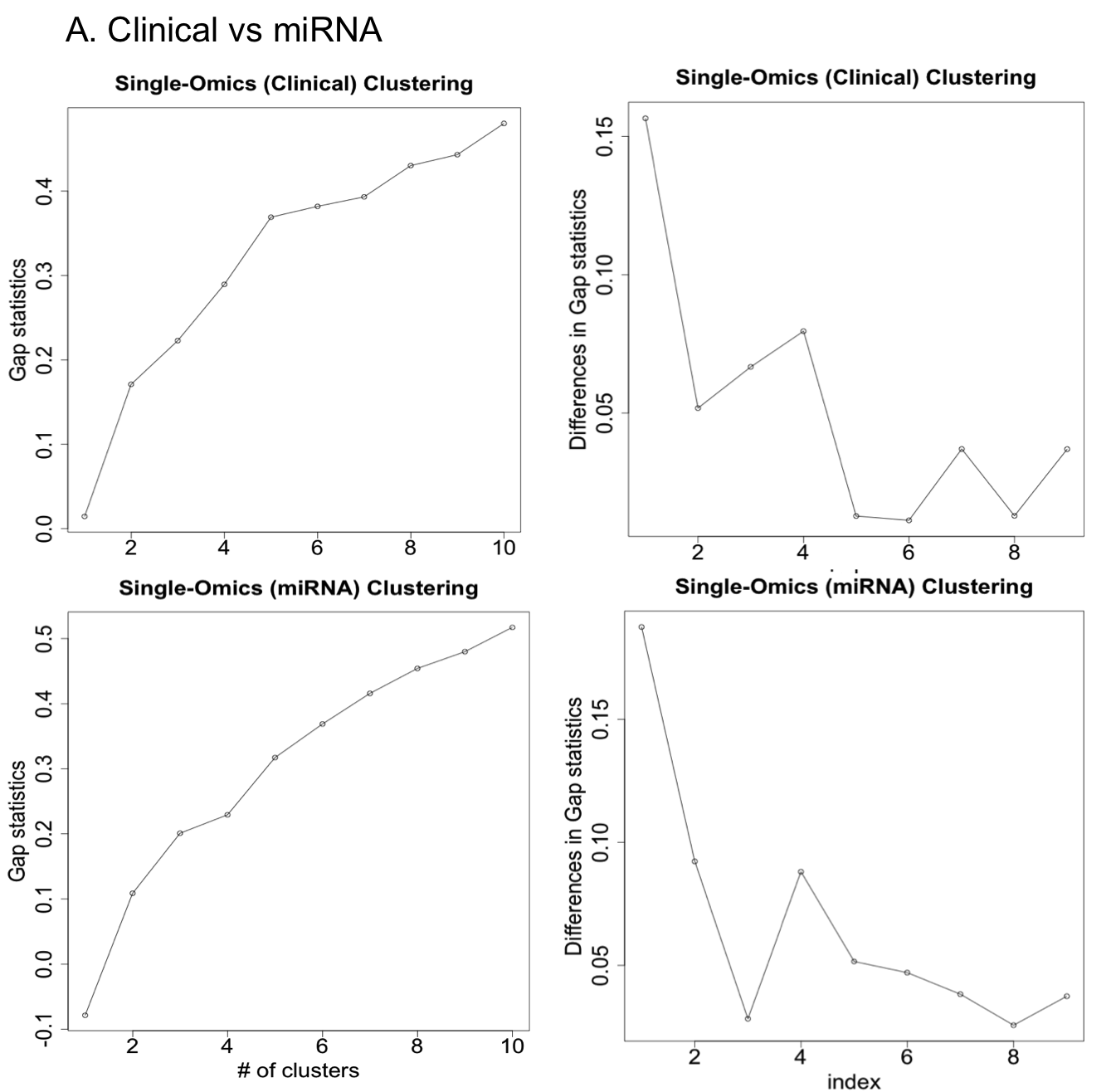


**Figure S9A:** The gap statistics and its scree plot to choose the optimal number of clustering (clinical and miRNA data).


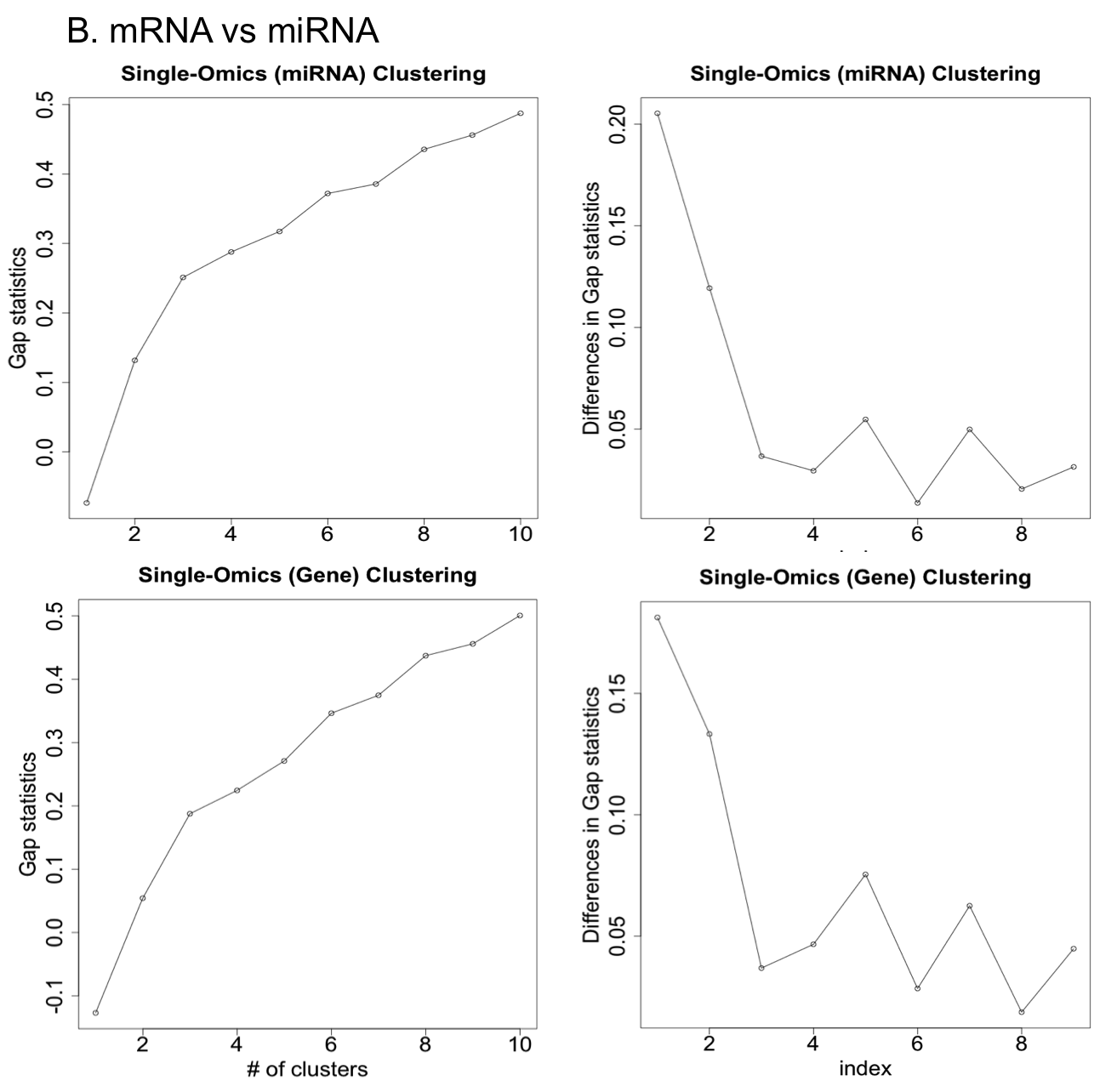


**Figure S9B:** The gap statistics and its scree plot to choose the optimal number of clustering (mRNA and miRNA data).


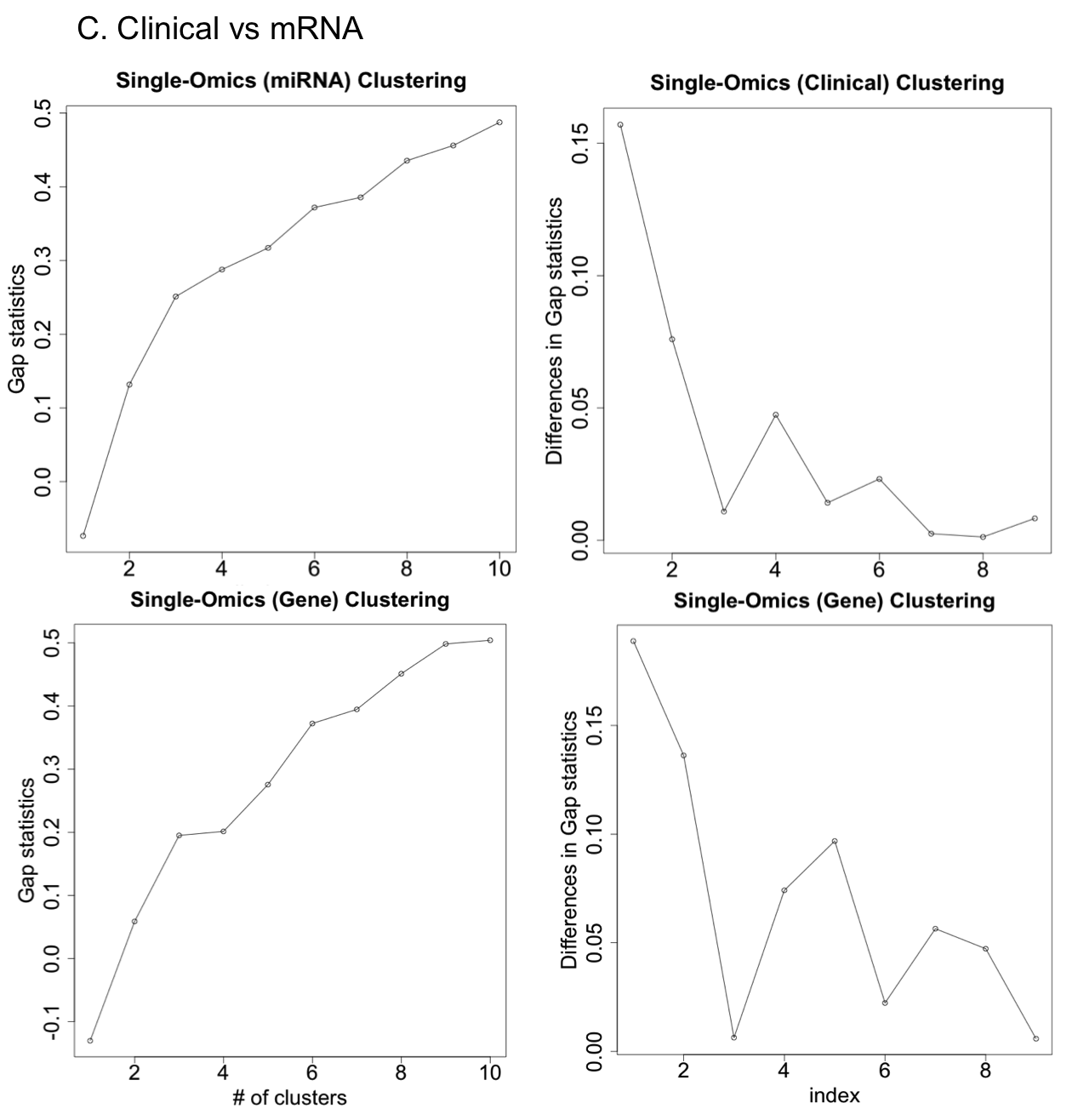


**Figure S9C:** The gap statistics and its scree plot to choose the optimal number of clustering (mRNA and clincal data).


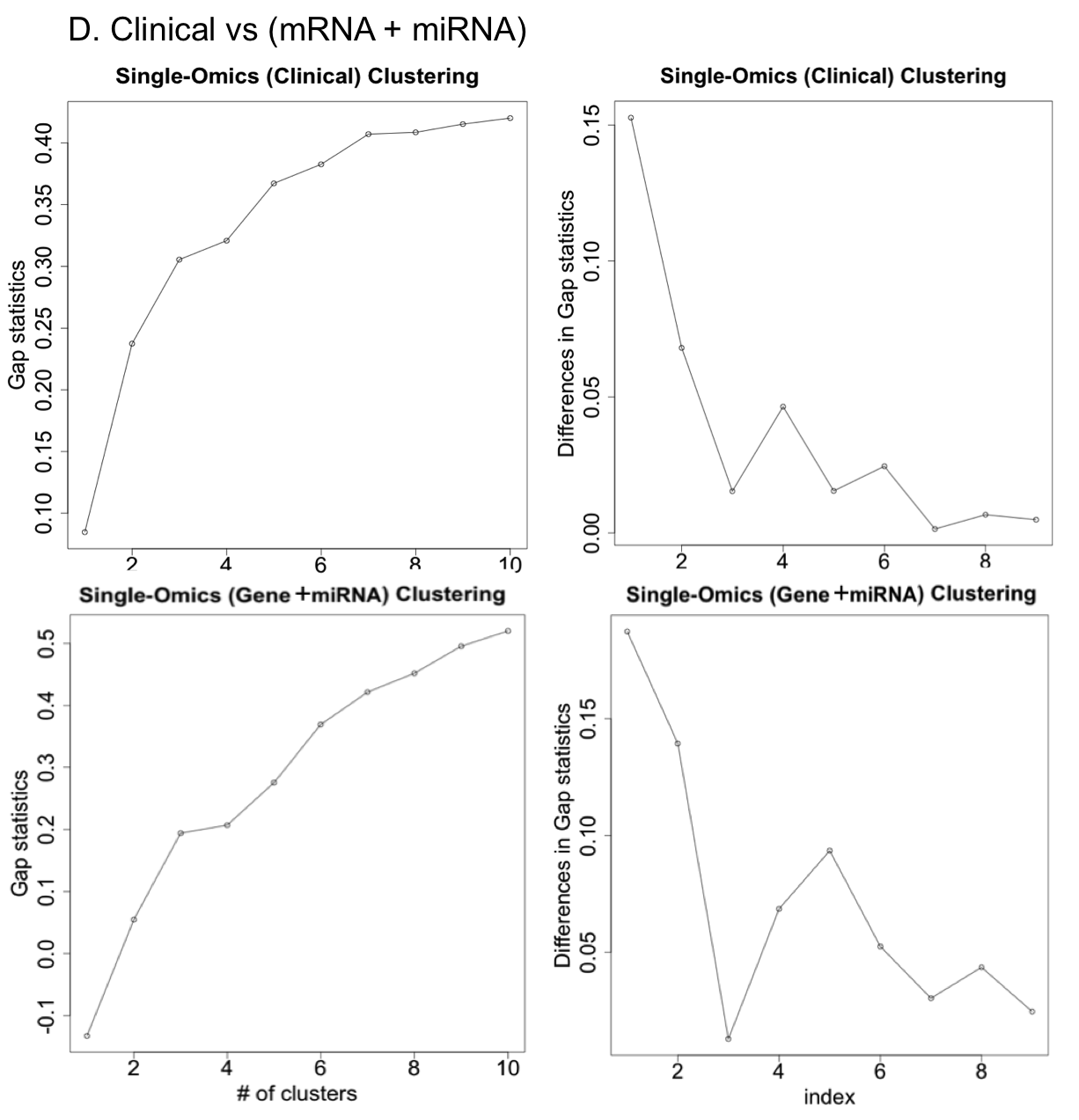


**Figure S9D:** The gap statistics and its scree plot to choose the optimal number of clustering (clincal data and combined data of mRNA and miRNA).


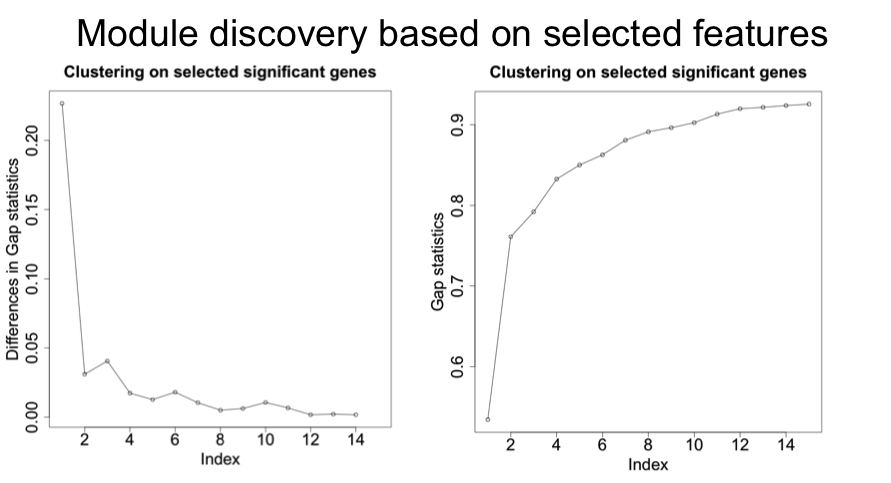

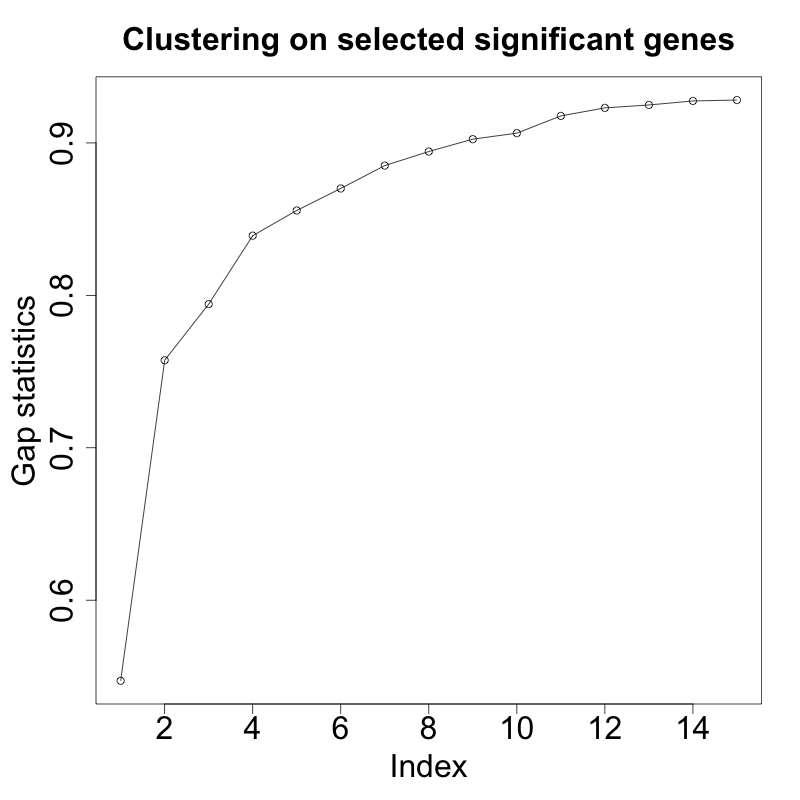

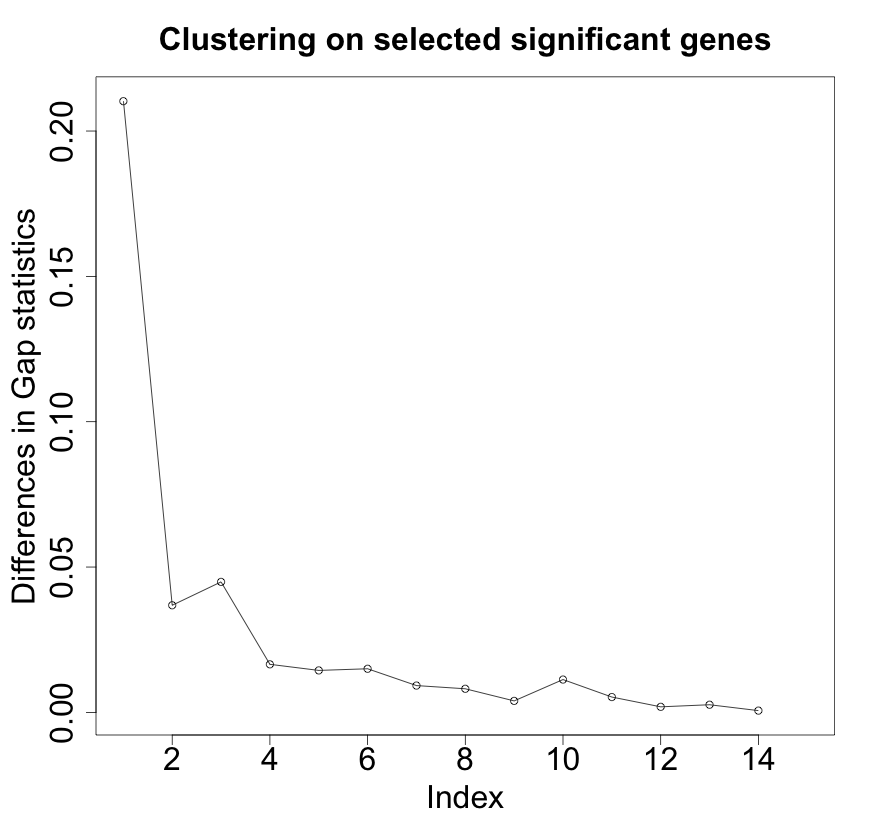


**Figure S10:** The best choice of the number of feature modules

**Figure S11:** Simulation study shows robust true feature discovery in “Feature Fusion”. The x-axis represents multiplication levels of noise features. The y-axis represents average ARIs from 100 simulations. Each figure is generated based on simulation scenarios of the different number of true features (e.g., 200, 400, and 600, respectively).


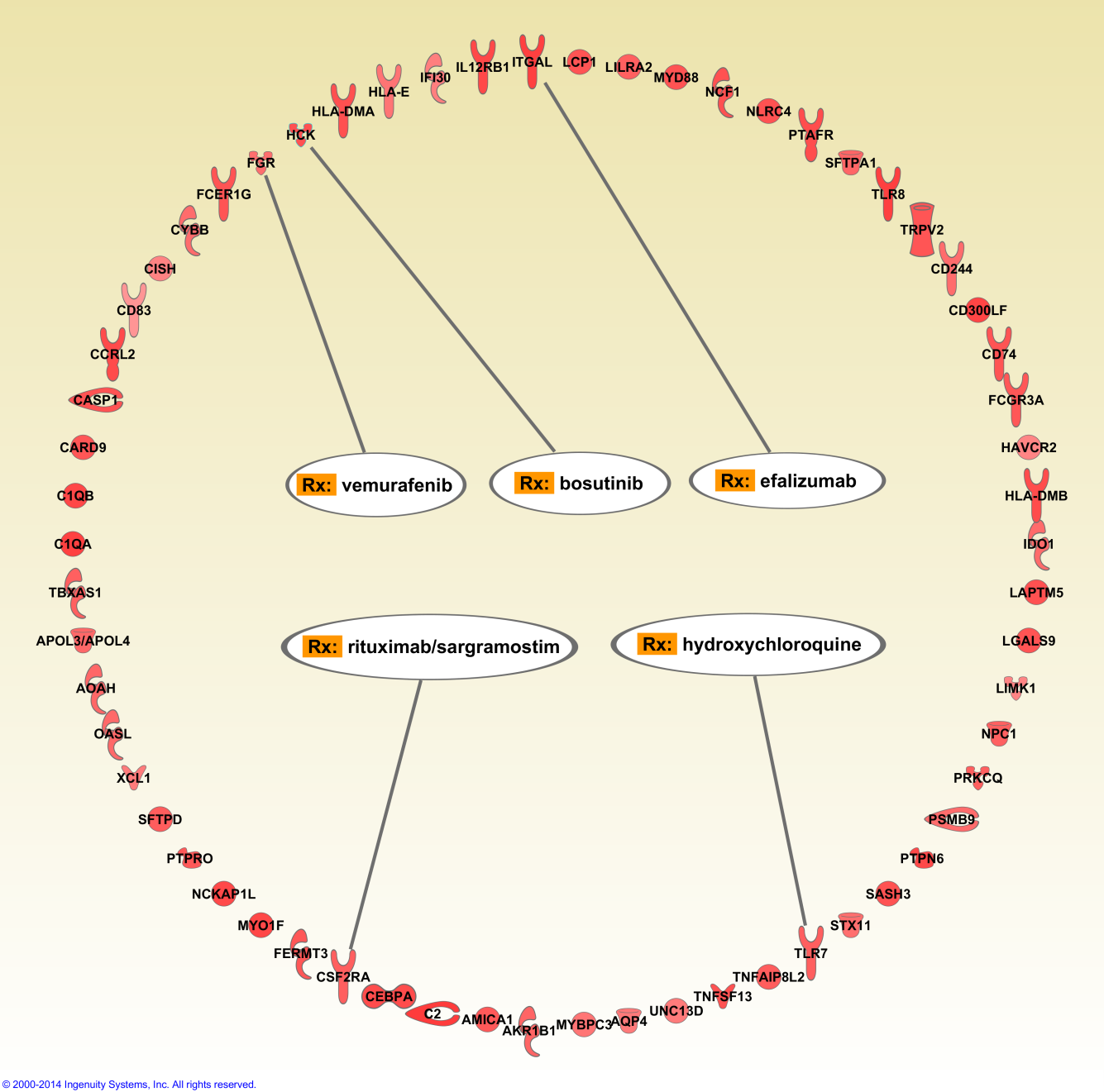


**Figure S12:** Immunomodulating drugs target overexpressed genes in module two

**Table S13:** The description of mRNA and miRNA lung disease data

**Table S14:** Various correlation types depending on variable attributes

**S15 Table:** The demographic summary of clinical features in each sub-cluster

**S16 Table:** Target gene enrichment analysis (via Fisher exact test) related to twelve significant miRNA features

**S17 Table:** Regression analysis on target miRNA features, and coefficient of determination


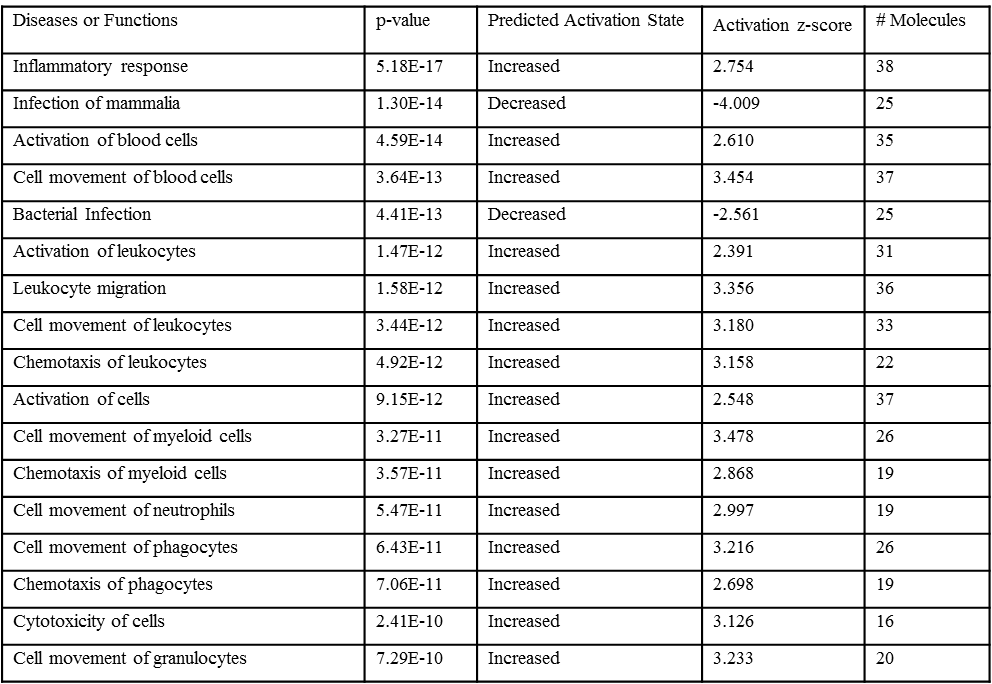


**S18 Table:** The top disease or functional annotations associated with genes in module two in Cluster E patients

*P value is calculated by the Fisher’s Exact Test. A P value threshold of ≤ 7.29E-10 was selected for statistical significance.

†The z-score predicts the direction of change for the function. An absolute z-score of ≥ 2 is considered significant. A function is: Increased if the z-score is ≥ 2 and decreased if the z-score is ≤ ‑2.

‡Predicted activation state is the predicted direction of change for the function based on the regulation z-score. Increased indicates that the z-score is ≥ 2 and decreased indicates that the z-score is ≤ ‑2.

A.

S13 T

B.

Cluster 3

Cluster 2

Cluster 1


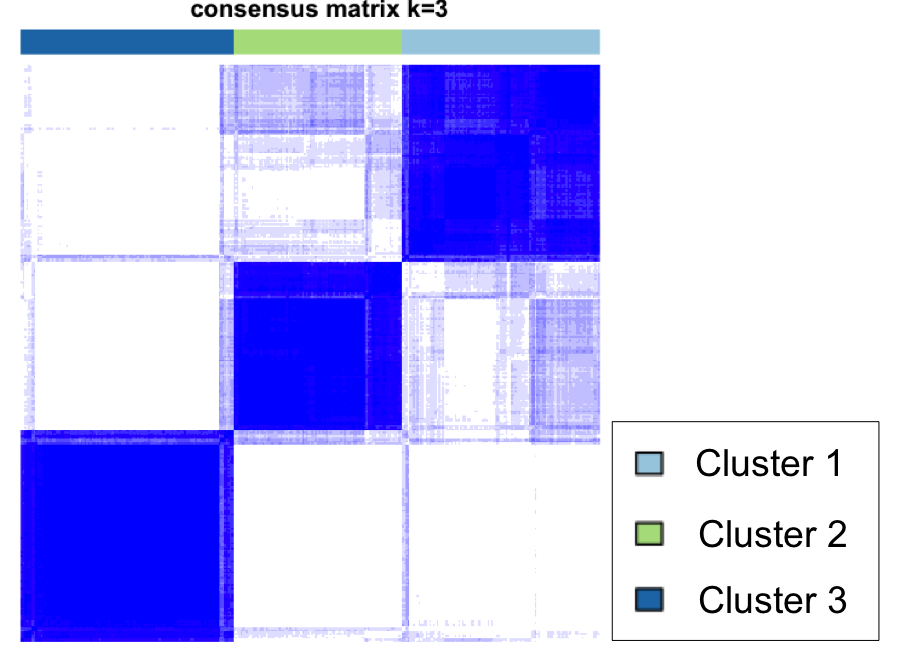
able

**S19 Figure:** Basic consensus clustering using only gene expression data. We performed basic consensus unsupervised clustering (i.e., used R-package “*ConsensusClusterPlus*”). Here we identify three consensus clusters notably correlated. However this consensus clustering does not clearly characterize a cluster as an “intermediate” group, just as we identified the sub-cluster E (Figure 4). Therefore the basic consensus clustering hardly identifies a novel sub-phenotype beyond the traditional disease definition.

**References**

1. Smyth GK, Speed T (2003) Normalization of cDNA microarray data. Methods 31: 265-273.

2. Qiu WL, Joe H (2006) Generation of random clusters with specified degree of separation. Journal of Classification 23: 315-334.

3. Hartigan JAaW, M. A. (1979) A K-means clustering algorithm. Applied Statistics 28, 100–108.

4. Witten D, Tibshirani R (2010) A Framework for Feature Selection in Clustering (vol 105, pg 713, 2010). Journal of the American Statistical Association 105: 1637-1637.

5. Fraley C, Raftery AE (2002) Model-based clustering, discriminant analysis, and density estimation. Journal of the American Statistical Association 97: 611-631.

6. Hubert L and Arabie P (1985). Comparing partitions. Journal of Classification 2 (1): 193–218.

7. Ronglai Shen SW, Qianxing Mo (2013) Sparse integrative clustering of multiple omics data sets. Annals of Applied Statistics 7: 269-294.

8. Wood SN (2003) Thin plate regression splines. J Roy Stat Soc B 65: 95-114.
