# Supplementary material for: Integrative phenotyping framework (iPF): integrative clustering of multiple omics data identifies novel lung disease subphenotypes
Source: BMC Genomics. 2015 Nov 11;16:924. doi: 10.1186/s12864-015-2170-4 (PMC4642618; doi:10.1186/s12864-015-2170-4)
Supplement: Additional file 4: — This is the first survey form to screen patients. Each colored label on the right hand side corresponds to variable names that appear in the clinical data set. (PDF 9214 kb) [file 12864_2015_2170_MOESM4_ESM.pdf]

LUNG TISSUE RESEARCH CONSORTIUM (LTRC)
SUBJECT SCREENING FORM

ID Number:	0	0		-					-	
Letter Code:										
Form Type:	S	S	0	1						

vis_dt

1. Date subject was enrolled: ___ ___ ___ - ___ ___ - 2 ___ ___ ___
Month Day Year

I. SCREENING INFORMATION

		Yes	No	
1.	Has the patient previously enrolled in the LTRC?	(1)	(2)	prvenrl

IF NO, go to question 2.

A. Date subject was last enrolled: ___ ___ ___ - ___ ___ - 2 ___ ___ ___ prve_dt
Month Day Year

B. Previous ID number in the LTRC: 0 0 __ - __ __ __ __ - __ prv_id

2. Inclusion criteria:

		Yes	No	
A.	Is the subject age 21 or above?	(1)	(2) STOP	age21
B.	Does the subject have a clinical indication of ILD leading to VATS or open lung biopsy?	(1)	(2)	ilddiag
C.	Does the subject have COPD leading to treatment with lung volume reduction surgery?	(1)	(2)	copdlvrs
D.	Does the subject have a clinical indication of ILD (including fibrosis, UIP, NSIP, or Sarcoidosis) or COPD as the principal reason for lung transplantation?	(1)	(2)	ipfcopd2
E.	Does the subject have a lung nodule/mass leading to resection?	(1)	(2)	lungmass
F	Clinical indication for lung surgery is ILD:	(1)	(2)	surgipf

3. Exclusion Criteria:

A.	Has the patient been diagnosed with an active primary infectious process (e.g. tuberculosis)?	(1) STOP*	(2)	priminf
B.	Is there a primary diagnosis of cystic fibrosis, berylliosis or pulmonary hypertension listed as the reason for a transplant?	(1) STOP*	(2)	excldia2

ID Number:	0	0		-					-	
Letter Code:										
Form Type:	S	S	0	1						

		Yes	No	
4.	Has the patient signed a consent form?	(1)	(2) STOP	consent

The patient is eligible if all of the following are true:

- **Questions 2A and 4 are checked YES**
- **At least 1 of questions 2B, C, D, or E is checked YES**
- **Both questions 3A and 3B are checked NO.**

5.	Is the patient eligible for the LTRC?	(1)	(2) STOP*	eligible

*If this is checked, the patient is not eligible for the LTRC.

II. ADMINISTRATIVE MATTERS

1. General Comments: ______________________________ gen_cmnt

____

2. Principal or Co-Investigator:

A. Signature: ______________________________ pi_sig

B. LTRC Staff No. ___ ___ ___ - ___ ___ ___ pi_no

3. Research Coordinator:

A. Signature: ______________________________ cert_sig

B. LTRC Staff No. ___ ___ ___ - ___ ___ ___ cert_no

4. Date Form Completed: ___ ___ ___ - ___ ___ - 2 ___ ___ ___ compl_dt

Month Day Year

LUNG TISSUE RESEARCH CONSORTIUM (LTRC)
DEMOGRAPHIC QUESTIONNAIRE

ID Number:	0	0		-					-	
Letter Code:										
Form Type:	D	Q	0	1						

vis_dt

1. Date of Interview: ___ ___ ___ - ___ ___ - 2 ___ ___ ___
Month Day Year

I will be asking some questions about your background.

I. DEMOGRAPHICS

1.	What is your birth date?	___ ___ ___ - ___ ___ - ___ ___ ___ ___ (Month Day Year)		birth_dt
2.	What is your gender?	Male	(1)	gender
		Female	(2)	
3.	What is your ethnicity?	Hispanic or Latino	(1)	ethnic
		Not Hispanic or Latino	(2)	
		Chooses Not to Disclose	(3)	

4. What race(s) do you primarily identify with (Check all that apply)?
INTERVIEWER READ LIST

A.	American Indian or Alaskan Native	(1)	race_a
B.	Asian	(1)	race_b
C.	Black or African American	(1)	race_c
D.	Native Hawaiian or other Pacific Islander	(1)	race_d
E.	White	(1)	race_e
F.	Chooses Not to Disclose	(1)	race_f

ID Number:	0	0		-					-	
Letter Code:										
Form Type:	D	Q	0	1						

II. ADMINISTRATIVE MATTERS

1. General comments: ______________________________ gen_cmnt

____

2. Research coordinator:

A. Signature: ______________________________ cert_sig

B. LTRC Staff No.: ___ ___ ___ - ___ ___ ___ cert_no

3. Date Form Completed: ___ ___ - ___ ___ - 2 ___ ___ ___ compl_dt

Month Day Year

LUNG TISSUE RESEARCH CONSORTIUM (LTRC)
CHANGE OF PROCEDURE/DEACTIVATION FORM

ID Number:	0	0		-					-	
Letter Code:										
Form Type:	D	F								

This form is to be completed when an expected procedure is not done or when a patient is deactivated from the study.

1. Date of report: __ __ - __ __ - 2 __ __ __ (Month Day Year) vis_dt

I. CHANGE OF PROCEDURE

If the patient has no procedure change, skip to Section II (i.e., this is a notice of deactivation only).

1. Interval for change of procedure (*check only one*) interval

Enrollment	(01)
6 Month	(02)
12 Month	(03)
18 Month	(04)
24 Month	(05)
30 Month	(06)
36 Month	(07)
42 Month	(08)
48 Month	(09)
54 Month	(10)
60 Month	(11)

2. Procedure (*check all that apply*)

	Missed		
A. Questionnaire(s)	(1)	Enrollment Only	misques
B. Cardiopulmonary Exercise Testing	(1)	Enrollment Only	miscpx
C. Six-Minute Walk Test	(1)		mis6mwlk
D. Pulmonary Function Testing	(1)		mispft
E. Laboratory Data	(1)		mislab
F. Research Blood Collection	(1)		misbld
G. CT Scan	(1)		misct
H. Concomitant Therapy	(1)		misther

For transplant patients, skip Item 2I.

	Not Collected	Delayed	
I. Tissue Collection	(1)	(2) Enrollment Only	mistiss

Non-transplant patients whose tissue collection is delayed for more than six months are expected to repeat procedures listed in 2C – 2H every six months until tissue is collected.

ID Number:	0	0		-					-	
Letter Code:										
Form Type:	D	F								

II. PATIENT DEACTIVATION

If the patient has not deactivated from the study, skip to Section III (i.e., this is only a notice of a change in procedure).

1. Date of deactivation: __ __ - __ __ - 2 __ __ __ (Month / Day / Year) wd_dt

2. Reason for deactivation (*select only one*) wd_reas
   - Subject is dead (1)
   - Subject is unwilling to participate (2)
   - Lost to follow-up (3)
   - Surgery Cancelled (4)
   - Other (5)

   A. Specify ______________________ wd_sp

3. Status of:

		Can be Used	Cannot Be Used	Not Collected	
A.	Tissue	(1)	(2)	(3)	stattis
B.	Blood	(1)	(2)	(3)	statbld
C.	CT Scan	(1)	(2)	(3)	statct

III. ADMINISTRATIVE MATTERS

1. General Comments: ______________________ gen_cmnt

____

2. Research Coordinator:

   A. Signature: ______________________ cert_sig

   B. LTRC Staff No. __ __ __ - __ __ __ cert_no

3. Date form completed: __ __ - __ __ - 2 __ __ __ (Month / Day / Year) compl_dt

LUNG TISSUE RESEARCH CONSORTIUM (LTRC)
MEDICAL HISTORY QUESTIONNAIRE

ID Number:	0	0		-					-	
Letter Code:										
Form Type:	M	H	0	1						

1. Date of Interview: ___ ___ ___ - ___ ___ - 2 ___ ___ ___ vis_dt
Month Day Year

I. PAST ILLNESSES

1. Now, I am going to read you a list of health problems. For each health problem, please tell me if you have ever had the problem.

		Yes	No	Don't Know	
A.	Angina	(1)	(2)	(3)	angina
B.	Heart failure (congestive heart failure or congestive heart disease)	(1)	(2)	(3)	chf
C.	Thromboembolic (blood clots in leg or lung)	(1)	(2)	(3)	thrmemb
D.	Arrhythmia (irregular heart beat)	(1)	(2)	(3)	arrhythm
E.	Hyperlipidemia (high cholesterol)	(1)	(2)	(3)	hylipid
F.	Renal Failure (kidney failure)	(1)	(2)	(3)	renfail
G.	Hepatitis (Liver infection or inflammation)	(1)	(2)	(3)	hepatis
H.	Cirrhosis or other serious, chronic liver disease	(1)	(2)	(3)	cirrhos
I.	Diabetes	(1)	(2)	(3)	diabetes
J.	HIV	(1)	(2)	(3)	hiv
K.	Lung Cancer	(1)	(2)	(3)	lungcanc
L.	Other Cancer (excluding basal cell carcinoma)	(1)	(2)	(3)	othcanc
	1) If YES, then specify: ______________________				canc_sp
M.	Rheumatoid Arthritis	(1)	(2)	(3)	arthrits
N.	Scleroderma	(1)	(2)	(3)	sclerdrm
O.	Lupus	(1)	(2)	(3)	lupus
P.	Polymyositis	(1)	(2)	(3)	polymyos
Q.	Other collagen vascular disease	(1)	(2)	(3)	colvasc
	1) If YES, specify: ______________________				vasc_sp

ID Number:	0	0		-					-	
Letter Code:										
Form Type:	M	H	0	1						

		Yes	No	Don't Know	
R.	Gastroesophageal Reflux Disease (GERD)	(1)	(2)	(3)	gerd
S.	Asthma	(1)	(2)	(3)	asthma

If NO, or DON'T KNOW, go to T.

1.	Was it confirmed by a doctor?	(1)	(2)	(3)	asthconf
T.	Pulmonary Hypertension	(1)	(2)	(3)	pulhyper
2.	Have you ever had attacks of bronchitis?	(1)	(2)	(3)	bronchev

If NO or DON'T KNOW, go to Question 3

A.	Was it confirmed by a doctor?	(1)	(2)	(3)	bronchcf

B. At what age was your first attack? bronchag __ __ Age in Years Don't Know (1) bronchdk

3.	Have you ever had respiratory failure requiring a ventilator?	(1)	(2)	respfail
4.	Have you unexpectedly lost a lot of weight in the past three months? (A lot is 10% or more of your body weight).	(1)	(2)	lostwt

5. Have you had any of the following surgical procedures?

A.	Tracheotomy/Tracheostomy	(1)	(2)	trach
B.	Bullectomy, pneumonectomy, or lobectomy (removal of all or part of the lung)/Prior surgical lung biopsy	(1)	(2)	lungsurg

If NO to B, go to D.

ID Number:	0	0		-					-	
Letter Code:										
Form Type:	M	H	0	1						

C. Which lung(s) and lobe(s) did you have the procedure on? (Please check all that apply.)

1)	Right Upper	(1)	surg_ru
2)	Right Middle	(1)	surg_rm
3)	Right Lower	(1)	surg_rl
4)	Left Upper	(1)	surg_lu
5)	Lingula	(1)	surg_lg
6)	Left Lower	(1)	surg_ll
7)	Don't Know	(1)	surg_dn

		Yes	No	
D.	Any other chest operations?	(1)	(2)	chestop
	1. If YES to D, Specify: ______________________			chsop_sp
6.	Have you ever had any chest injuries?	(1)	(2)	chestinj
	A. If YES to Question 6, Specify: ______________________			chsin_sp

II. CURRENT ILLNESSES

1. Has a doctor told you that you have any of the following?

		Yes	No	Don't Know	
A.	Chronic Obstructive Pulmonary Disease (COPD)	(1)	(2)	(3)	copd_cur
B.	Chronic Bronchitis	(1)	(2)	(3)	brnc_cur
C.	Emphysema	(1)	(2)	(3)	emph_cur
D.	Asthma	(1)	(2)	(3)	asth_cur

		Yes	No	Don't Know	
2.	Do you have alpha-1 antitrypsin deficiency?	(1)	(2)	(3)	a1adefc
3.	Has a doctor told you that you have a fibrotic lung disease?	(1)	(2) ↓	(3) ↓	fibrldis

If NO or DON'T KNOW, go to Part III.

		Yes	No	Don't Know	
4.	Was the fibrotic lung disease documented by surgical biopsy?	(1)	(2)	(3)	flddoc

**ID Number:**	0	0		-					-	
**Letter Code:**										
**Form Type:**	M	H	0	1						

III. ADMINISTRATIVE MATTERS

1. General Comments: ______________________________________ gen_cmnt

   ____

2. Research Coordinator:

   A. Signature: ______________________________________ cert_sig

   B. LTRC Staff No. ___ ___ ___ - ___ ___ ___ cert_no

3. Date form completed: ___ ___ - ___ ___ - 2 ___ ___ ___ compl_dt

   Month Day Year

LUNG TISSUE RESEARCH CONSORTIUM (LTRC)
FAMILY HISTORY QUESTIONNAIRE

ID Number:	0	0		-					-	
Letter Code:										
Form Type:	F	H	0	1						

1. Date of Interview: ___ ___ ___ - ___ ___ - 2 ___ ___ ___ vis_dt
Month Day Year

GENERAL INSTRUCTIONS: ASK PARTICIPANT ALL QUESTIONS, SKIPPING OVER SECTIONS WHEN APPROPRIATE.

Beginning Script:
The following questions have to do with your blood relatives (for example your birth mother, your birth father, brother, sister, child). Please answer these questions to the best of your knowledge. If you are unsure of any of the answers, please respond with "I don't know". Remember, these questions are about blood relatives.

I. FIRST DEGREE BLOOD RELATIVES

1. Do you know who at least one of your birth parents are? Yes (1) No (2) parents

   Please answer NO if you only know about your adoptive, foster or step-parents.

2. How many blood siblings do you have (include half siblings)? siblings ___ ___ Unknown (1) sib_unk

3. How many children do you have? children ___ ___ (1) childunk

IF ITEM 1 IS YES OR ITEM 2 OR 3 HAS A NUMBER GREATER THAN ZERO, ASK ALL REMAINING QUESTIONS.

ID Number:	0	0		-					-	
Letter Code:										
Form Type:	F	H	0	1						

		Yes	No	Unknown	
4.	Have any of your first degree blood relatives (parent, sibling, child) developed any of the following?				
	A. COPD	(1)	(2)	(3)	rel_copd
	B. Chronic bronchitis	(1)	(2)	(3)	rel_bron
	C. Emphysema	(1)	(2)	(3)	rel_emph
	D. Asthma	(1)	(2)	(3)	rel_asth
5.	Have any of your first degree blood relatives (parent,sibling, child) had alpha-1 antitrypsin deficiency?	(1)	(2)	(3)	rel_a1ad
6.	Have any of your first degree blood relatives (parent, sibling, child) developed a fibrotic lung disease?	(1)	(2) ↓	(3) ↓	rel_fld

IF NO OR UNKNOWN, GO TO QUESTION 8.

		Yes	No	Unknown	
7.	Was a fibrotic lung disease documented by biopsy in any of these relatives?	(1)	(2)	(3)	rel_biop
8.	Have any of your first degree blood relatives had any of the following?				
	A. Rheumatoid arthritis	(1)	(2)	(3)	rel_arth
	B. Scleroderma	(1)	(2)	(3)	rel_sclr
	C. Lupus	(1)	(2)	(3)	rel_lups
	D. Polymyositis	(1)	(2)	(3)	rel_pmyo
	E. Other collagen vascular disease	(1)	(2)	(3)	rel_ovas
	1) If YES to E, specify: ______________________				rel_sp
	F. Pulmonary Hypertension	(1)	(2)	(3)	rel_phyp

ID Number:	0	0		-					-	
Letter Code:										
Form Type:	F	H	0	1						

II. ADMINSTRATIVE MATTERS

1. General Comments: ______________________________________ gen_cmnt

   ____

2. Research Coordinator:

   A. Signature: ______________________________________ cert_sig

   B. LTRC Staff No. __ __ __ - __ __ __ cert_no

3. Date Form Completed: __ __ __ - __ __ - 2 __ __ __ compl_dt
   Month Day Year

LUNG TISSUE RESEARCH CONSORTIUM
SMOKING HISTORY FORM

ID Number:	0	0		-					-	
Letter Code:										
Form Type:	S	H	0	1						

1. Date of Interview: ___ ___ ___ - ___ ___ - 2 ___ ___ ___ vis_dt
Month Day Year

I. SMOKING HISTORY

1. CIGARETTES

A. Have you ever smoked at least 100 cigarettes in your lifetime? (not cigars or pipes) — Yes (1) No (2) ↓ smokcig

IF NO, GO TO 2A.

B. Do you now smoke cigarettes? — Yes (1) No (2) smoknow

C. When smoking cigarettes, what is the average number of cigarettes you smoked per day? ___ ___ ___ cigarettes per day cignum

D. On average, how many years in total have you smoked cigarettes? ___ ___ Years cigyrs

If you are a current smoker, skip E and go to 2A.

E. When did you stop smoking cigarettes? cstopmon ___ ___ ___ - cstopyr ___ ___ ___ ___
Month Year

2. CIGARS/CIGARILLOS/PIPES

A. Have you ever smoked at least 100 cigars, cigarillos or pipes in your lifetime? — Yes (1) No (2) smokoth

IF NO, GO TO 3A.

ID Number:	0	0		-					-	
Letter Code:										
Form Type:	S	H	0	1						

B. Do you now smoke cigars, cigarillos or pipes? Yes (1) No (2) smoknow2

C. When smoking, what is the average number of cigars, cigarillos or pipe bowls you smoke in a day? smoknum

- Less than one (1)
- one – two daily (2)
- three – four daily (3)
- five – seven daily (4)
- Eight or more daily (5)

D. On average, how many years in total have you smoked cigars, cigarillos, or pipes? __ __ Years smokyrs

If you are a current smoker, skip E and go to 3A.

E. When did you stop smoking cigars, cigarillos, or pipes? ostopmon ostopyr __ __ __ - __ __ __ __ Month Year

3. PASSIVE SMOKE EXPOSURE

	Yes	No	Don't Know	
A. Have you ever lived in a household in which people smoked?	(1)	(2)	(3)	smokhous
B. Have you ever worked in an environment with significant second-hand smoke exposure?	(1)	(2)	(3)	smokwork

If NO or DON'T KNOW to A and B, go to D.

	≤ 10 Yrs	> 10 Yrs	
C. How long were you exposed to second-hand smoke in your home or work environment?	(1)	(2)	smoklen

	Yes	No	Don't Know	
D. Did your mother smoke while she was pregnant with you?	(1)	(2)	(3)	momsmok

ID Number:	0	0		-					-	
Letter Code:										
Form Type:	S	H	0	1						

II. ADMINISTRATIVE MATTERS

1. General Comments: ____ gen_cmnt

____

2. Research Coordinator:

A. Signature: ____ cert_sig

B. LTRC Staff Number: ___ ___ ___ - ___ ___ ___ cert_no

3. Date Form Completed: ___ ___ - ___ ___ - 2 ___ ___ ___ compl_dt

Month Day Year

LUNG TISSUE RESEARCH CONSORTIUM (LTRC)
CONCOMITANT THERAPY FORM

ID Number:	0	0		-					-	
Letter Code:										
Form Type:	C	T								

1. Date of Interview: __ __ __ - __ __ - 2 __ __ __ (Month, Day, Year) vis_dt

I. SPECIFIC MEDICAL TREATMENT INFORMATION

1. Have you taken the following medications in the 30 days prior to today?

	Yes	No	
A. Systemic (Oral or IV) corticosteroids (e.g. prednisone, Medrol)	(1)	(2)	ssteroid
B. Interferon (gamma or beta)	(1)	(2)	interfn
C. Immune suppressive agents (such as cyclophosphamide, azathioprine, mycophenylate, TNF-alpha antagonists, methotrexate, or other immune suppressive agents or investigational drugs)	(1)	(2)	immsupp

2. Have you ever taken the following medications? If YES, please specify the duration (number of years) of use and how long ago (in years) you stopped.

		(1) Ever Yes	(1) Ever No	(2) Duration Number of Years	(3) How Long ago stopped
A. Amiodarone	amio_ev	(1)	(2)	amio_dur	amio_stp
B. Nitrofurantoin	nitr_dv	(1)	(2)	nitr_dur	nitr_stp
C. Chemotherapy for cancer (such as Bleomycin, cyclophosphamide, ARA-C, Nitrosoureas, Gemcytibine, Imuran, Iressa)	chem_ev	(1)	(2)	chem_dur	chem_stp
D. Thoracic Radiation Therapy for Malignancy	trad_ev	(1)	(2)	trad_dur	trad_stp

ID Number:	0	0		-					-	
Letter Code:										
Form Type:	C	T								

II. OTHER MEDICATION INFORMATION

			Yes	No	
1.	Have you taken any inhaled steroids (e.g. Flovent, Pulmicort, Aerobid, Advair) in the last 30 days?		(1)	(2)	isteroid
2.	Have you taken any of the following types of bronchodilator medication in the last 30 days?				
	A.	Inhaled beta-agonists such as Serevent, salmeterol, Ventolin, Proventil, Albuterol, Foradil	Yes (1)	No (2)	bronchd1
	B.	Anticholinergics such as Atrovent, Combivent, Spiriva	(1)	(2)	bronchd2
	C.	Oral beta-agonists, such as Brethaire, Ventolin, Proventil.	(1)	(2)	bronchd3
	D.	Theophylline	(1)	(2)	bronchd4
	E.	Other	(1)	(2)	bronchd5
		1) If YES to E, specify: ____________________			brond_sp

3. Have you taken any of the following types of medications within the last 30 days? If YES, please specify the duration (number of years) of use.

			(1) In The Last 30 Days Yes	No	(2) Duration Number of years	
A.	Ace Inhibitor	aceinhib	(1)	(2)	__ __ . __	ace_dur
B.	Statins	statins	(1)	(2)	__ __ . __	stat_dur
C.	Macrolides	macrolid	(1)	(2)	__ __ . __	macr_dur
D.	Cox-2 Inhibitors	cox2inhb	(1)	(2)	__ __ . __	cox2_dur
E.	Ketaconazole	ketacon	(1)	(2)	__ __ . __	keta_dur

ID Number:	0	0		-					-	
Letter Code:										
Form Type:	C	T								

III. ADMINISTRATIVE MATTERS

1. General Comments: ____ gen_cmnt

   ____

2. Research Coordinator:

   A. Signature: ______________________________________ cert_sig

   B. LTRC Staff No: ___ ___ ___ - ___ ___ ___ cert_no

3. Date form completed: ___ ___ ___ - ___ ___ - 2 ___ ___ ___ compl_dt
   Month Day Year

LUNG TISSUE RESEARCH CONSORTIUM (LTRC)

SYMPTOM QUESTIONNAIRE

ID Number:	0	0		-					-	
Letter Code:										
Form Type:	S	Q	0	1						

1. Date of Interview: ___ ___ - ___ ___ - _2_ _ _ _ _ ___ vis_dt
Month Day Year

I. COUGH

These questions pertain mainly to your chest. Please answer YES or NO. If you are in doubt about whether your answer is YES or NO, then answer NO.

		Yes	No	
1.	Do you usually have a cough?	(1)	(2) ↓	cough
	(Count a cough with first smoke or on first going out-of-doors). Exclude clearing your throat.	**If NO, go to Question 3.**		
2.	Do you usually cough as much as 4 to 6 times a day, 4 or more days out of the week?	(1)	(2)	coughdy
3.	Do you usually cough at all on getting up, or first thing in the morning?	(1)	(2)	cougham
4.	Do you usually cough at all during the rest of the day or night?	(1) ↓	(2) ↓	coughpm
		**If YES to any of questions 1-4, go to Question 5. If NO to all questions, go to Part II.**		
5.	Do you usually cough like this on most days for three consecutive months during the year?	(1)	(2)	coughmo
6.	For how many years have you had this cough?	___ ___ Years		coughyrs

ID Number:	0	0		-					-	
Letter Code:										
Form Type:	S	Q	0	1						

II. PHLEGM

		Yes	No	
1.	Do you usually bring up phlegm from your chest?	(1)	(2) ↓	phlegm

Count phlegm with the first smoke or on first going out-of-doors. Exclude phlegm from the nose. Count swallowed phlegm.

If NO, go to Question 3.

		Yes	No	
2.	Do you usually bring up phlegm like this as much as twice a day, 4 or more days out of the week?	(1)	(2)	phlegmdy
3.	Do you usually bring up phlegm at all on getting up, or first thing in the morning?	(1)	(2)	phlegmam
4.	Do you usually bring up phlegm at all during the rest of the day or at night?	(1) ↓	(2) ↓	phlegmpm

If YES to any of questions 1-4, go to Question 5. If NO to all, go to Part III.

		Yes	No	
5.	Do you bring up phlegm like this on most days for three consecutive months or more during the year?	(1)	(2)	phlegmmo
6.	For how many years have you had trouble with phlegm?	__ __ Years		phlegmyr

III. EPISODES OF COUGH AND PHLEGM

If question 1 in Part I was answered NO, go to Part IV.

		Yes	No	
1.	Have you had periods or episodes of increased cough and phlegm lasting for three weeks or more each year?	(1)	(2) ↓	epiwks

If NO, go to Part IV.

		epiyrs	epiyr_dk
2.	For how long have you had at least one such episode per year?	__ __ Years	Don't Know (1)

ID Number:	0	0		-					-	
Letter Code:										
Form Type:	S	Q	0	1						

IV. WHEEZING

		Yes	No	
1.	Does your chest ever sound wheezy or whistling?			
	A. When you have a cold?	(1)	(2)	cold
	B. Occasionally, apart from colds?	(1)	(2)	nocold
	C. Most days or nights?	(1)	(2) ↓	wheeze

If NO, to all of the above, go to Question 3.

2.	For how many years has this been present?	__ __ Years		wheezyrs
3.	Have you ever had an attack of wheezing that has made you feel short of breath?	(1)	(2) ↓	shrtbr

If NO, go to Part V.

4.	How old were you when you had your first attack?	__ __ Years		shrtbrag
5.	Have you had two or more such episodes?	(1)	(2)	shrtbr2
6.	Have you ever required medicine or treatment for the(se) attack(s)?	(1)	(2)	shrtbrrx
7.	Have you had an attack of wheezing that has made you feel short of breath in the past year?	(1)	(2) ↓	shrtbryr

If NO, go to Part V.

8.	Have you had two or more such episodes in the past year?	(1)	(2)	shrbryr2
9.	Have you required medicine or a treatment for the(se) attack(s) in the past year?	(1)	(2)	shrbrrx2

V. BREATHLESSNESS

		Yes	No	
1.	Are you disabled from walking by any condition OTHER than heart or lung disease?	(1)	(2)	cantwalk

If YES, please describe the nature of the condition(s): walk_sp

A. ______________________________

____

____

____

ID Number:	0	0		-					-	
Letter Code:										
Form Type:	S	Q	0	1						

2. The following questions are designed to determine how much work would make you short of breath. Please answer each question. If you use supplemental oxygen please answer each question as though you are NOT using your oxygen.

		Yes	No	
A.	Are you troubled by shortness of breath when hurrying on the level or walking up a slight hill?	(1)	(2)	shrbrhil

If NO to A, go to Part VI.

		Yes	No	
B.	Do you have to walk slower than people of your age on the level because of breathlessness?	(1)	(2)	walkslow
C.	Do you ever have to stop for breath when walking at your own pace on the level?	(1)	(2)	walkstop
D.	Do you ever have to stop for breath after walking about 100 yards (or after a few minutes) on the level?	(1)	(2)	walkdist
E.	Are you too breathless to leave the house or breathless on dressing or undressing?	(1)	(2)	cantleav

VI. CHEST COLDS AND CHEST ILLNESSES

1. How often do you get colds? oftcold

Less often than once a year	(1)
Once a year	(2)
2-4 times per year	(3)
5 or more times per year	(4)

If LESS THAN ONCE A YEAR, go to Question 5.

		Yes	No	
2.	Do your colds usually go to your chest? ("Usually" means more than half the time).	(1)	(2)	chstcold

3. How often did you get colds in the past 12 months? oftcold2

Not at all	(1)
Once	(2)
2-4 times	(3)
5 or more times	(4)

If NOT AT ALL, go to Question 5.

**ID Number:**	0	0		-					-	
**Letter Code:**										
**Form Type:**	S	Q	0	1						

4. Did your colds in the past 12 months usually go to your chest? ("Usually" means more than half the time). Yes (1) No (2) chstoft

5. During the past 12 months, have you had any chest illnesses that have kept you off work, indoors at home, or in bed? Yes (1) No (2) chstyr

   If NO, go to Question 8.

6. Did you produce phlegm with any of these chest illnesses? (1) (2) chstphlm

7. In the past 12 months, how many such illnesses, with (increased*) phlegm, did you have which lasted a week or more? ___ ___ No. of Illnesses chstnbr
   *(for persons who usually have phlegm)

8. Did you have any lung trouble before the age of 16? Yes (1) No (2) lung16

9. Did you have any chest illness before the past 12 months? (1) (2) chsill12

   A. If YES to question 9, specify: ______________________ chill_sp

VII. ADMINISTRATIVE MATTERS

1. General Comments: ______________________ gen_cmnt
   ____

2. Research Coordinator:

   A. Signature: ______________________ cert_sig

   B. LTRC Staff Number: ___ ___ ___ - ___ ___ ___ cert_no

3. Date Form Completed: ___ ___ ___ - ___ ___ - 2 ___ ___ ___ compl_dt
   Month Day Year

LUNG TISSUE RESEARCH CONSORTIUM (LTRC)
SF-12 HEALTH SURVEY

ID Number:	0	0		-					-	
Letter Code:										
Form Type:	H	S	0	1						

1. Date of Interview: ___ ___ ___ - ___ ___ - 2 ___ ___ ___ vis_dt
   Month Day Year

I. EVALUATION

General Instructions: This survey asks for your views about your health. This information will help keep track of how you feel and how well you are able to do your usual activities.

For each of the following questions, please mark the space that best describes your answer.

1. In general, would you say your health is: gen_hlth

Excellent	Very Good	Good	Fair	Poor
(1)	(2)	(3)	(4)	(5)

2. The following questions are about activities you might do during a typical day. Does your health now limit you in these activities? If so, how much?

	Yes, limited a lot	Yes, limited a little	No, not limited at all
A. Moderate activities such as moving a table, pushing a vacuum cleaner, bowling or playing golf (phyftn1)	(1)	(2)	(3)
B. Climbing several flights of stairs (phyftn2)	(1)	(2)	(3)

3. During the past 4 weeks, how much of the time have you had any of the following problems with your work or other regular daily activities as a result of your physical health?

	All of the time	Most of the time	Some of the time	A little of the time	None of the time
A. Accomplished less than you would like (phyhlt1)	☐ (1)	☐ (2)	☐ (3)	☐ (4)	☐ (5)
B. Were limited in the kind of work or other activities (phyhlt2)	☐ (1)	☐ (2)	☐ (3)	☐ (4)	☐ (5)

4. During the past 4 weeks, how much of the time have you had any of the following problems with your work or other regular daily activities as a result of any emotional problems (such as feeling depressed or anxious)?

	All of the time	Most of the time	Some of the time	A little of the time	None of the time
A. Accomplished less than you would like (emotpb1)	☐ (1)	☐ (2)	☐ (3)	☐ (4)	☐ (5)
B. Did work or other activities less carefully than usual (emotpb2)	☐ (1)	☐ (2)	☐ (3)	☐ (4)	☐ (5)

ID Number:	0	0		-					-	
Letter Code:										
Form Type:	H	S	0	1						

5. During the past 4 weeks, how much did pain interfere with your normal work (including both work outside the home and housework)? painwrk

Not at all	A little bit	Moderately	Quite a bit	Extremely
▼ (1)	▼ (2)	▼ (3)	▼ (4)	▼ (5)

6. These questions are about how you feel and how things have been with you during the past 4 weeks. For each question, please give the one answer that comes closest to the way you have been feeling. How much of the time during the past 4 weeks . . .

	All of the time	Most of the time	Some of the time	A little of the time	None of the time	
	▼	▼	▼	▼	▼	
A. Have you felt calm and peaceful?	(1)	(2)	(3)	(4)	(5)	anxity1
B. Did you have a lot of energy?	(1)	(2)	(3)	(4)	(5)	anxity2
C. Have you felt downhearted and depressed?	(1)	(2)	(3)	(4)	(5)	anxity3

7. During the past 4 weeks, how much of the time has your physical health or emotional problems interfered with your social activities (like visiting friends, relatives, etc)? hltsact

All of the time	Most of the time	Some of the time	A little of the time	None of the time
▼ (1)	▼ (2)	▼ (3)	▼ (4)	▼ (5)

II. ADMINISTRATIVE MATTERS

1. General Comments: ____ gen_cmnt

____

2. Research Coordinator:

   A. Signature: ____ cert_sig

   B. LTRC Staff Number: ___ ___ ___ - ___ ___ ___ cert_no

3. Date Form Completed: ___ ___ ___ - ___ ___ - 2 ___ ___ ___ compl_dt
   Month Day Year

LUNG TISSUE RESEARCH CONSORTIUM (LTRC)
ST. GEORGE'S RESPIRATORY QUESTIONNAIRE

ID Number:	0	0		-					-	
Letter Code:										
Form Type:	R	Q	0	1						

1. Date of Interview: ___ ___ ___ - ___ ___ - 2 ___ ___ ___ vis_dt
Month Day Year

I. EVALUATION

This questionnaire is designed to help us learn much more about how your breathing is troubling you and how it affects your life. We are using it to find out which aspects of your illness cause you the most problems, rather than what the doctors and nurses think your problems are.

Please read the questions carefully and ask if you do not understand anything. Do not spend too long deciding about your answers.

Please describe how often your lung/respiratory problems have affected you over the last four weeks.

Part 1: Four week description. Please describe how often your lung/respiratory problems have affected you over the last four weeks. Please check one answer for each question.

1. Over the last 4 weeks, I have coughed: cgh4wk

Almost every day	(1)
Several days a week	(2)
A few days a month	(3)
Only with lung/respiratory infections	(4)
Not at all	(5)

2. Over the last 4 weeks, I have brought up phlegm (sputum): phlm4wk

Almost every day	(1)
Several days a week	(2)
A few days a month	(3)
Only with lung/respiratory infections	(4)
Not at all	(5)

3. Over the last 4 weeks, I have had shortness of breath: shrtb4wk

Almost every day	(1)
Several days a week	(2)
A few days a month	(3)
Only with lung/respiratory infections	(4)
Not at all	(5)

ID Number:	0	0		-					-	
Letter Code:										
Form Type:	R	Q	0	1						

4. Over the last 4 weeks, I have had episodes of wheezing: wheez4wk

Almost every day	(1)
Several days a week	(2)
A few days a month	(3)
Only with lung/respiratory infections	(4)
Not at all	(5)

5. During the last 4 weeks, how many severe or very unpleasant episodes of lung/respiratory problems have you had: resp4wk

More than three episodes	(1)
Three episodes	(2)
Two episodes	(3)
One episode	(4)
No episodes	(5)

6. How long did the worst episode of lung/respiratory problem last:
(If you reported NO EPISODES of lung/respiratory problems in Question 5, go to question 7.) resplen

A week or more	(1)
Three or more days	(2)
One or two days	(3)
Less than a day	(4)

7. Over the last 4 weeks, in an average week, how many good days (with few lung/respiratory problems) have you had: gddy4wk

None	(1)
One or two	(2)
Three or four	(3)
Nearly every day	(4)
Every day	(5)

8. If you wheeze, is it worse in the morning?
(If you do not wheeze, go to the next question.) wheezam

No	(1)
Yes	(2)

ID Number:	0	0		-					-	
Letter Code:										
Form Type:	R	Q	0	1						

Part 2 – Section 1

9. How would you describe your lung/respiratory condition (check one only): respdesc

The most important problem I have	(1)
Causes me quite a lot of problems	(2)
Causes me a few problems	(3)
Causes no problem	(4)

10. If you have ever held a job, please check one of these: respjob

My lung/respiratory problem made me stop my job	(1)
My lung/respiratory problem interferes with my job or made me change my job	(2)
My lung/respiratory problem does not affect my job	(3)

Section 2: These are questions about what activities usually make you feel short of breath. Please check either True or False as it applies to you now.

		True	False	
11.	Sitting or lying still:	(1)	(2)	sbact1
12.	Washing yourself or dressing:	(1)	(2)	sbact2
13.	Walking in the house:	(1)	(2)	sbact3
14.	Walking outside on level ground:	(1)	(2)	sbact4
15.	Walking up a flight of stairs:	(1)	(2)	sbact5
16.	Walking up hills:	(1)	(2)	sbact6
17.	Playing sports or active games (baseball, tennis, etc.):	(1)	(2)	sbact7

ID Number:	0	0		-					-	
Letter Code:										
Form Type:	R	Q	0	1						

Section 3: These are more questions about your cough and shortness of breath. Please check either True or False as it applies to you now.

		True	False	
18.	Coughing hurts:	(1)	(2)	cghhurt
19.	Coughing makes me tired:	(1)	(2)	cghtire
20.	I am short of breath when I talk:	(1)	(2)	sbtalk
21.	I am short of breath when I bend over:	(1)	(2)	sbbend
22.	My coughing or breathing disturbs my sleep:	(1)	(2)	sbsleep
23.	I become exhausted easily:	(1)	(2)	exhstd

Section 4: These are questions about other effects that your lung/respiratory problems may have on you. Please check either True or False as it applies to you now.

		True	False	
24.	My coughing or breathing is embarrassing in public:	(1)	(2)	respeff1
25.	My lung/respiratory problem is a nuisance to my family, friends, or neighbors:	(1)	(2)	respeff2
26.	I panic or get afraid when I cannot catch my breath:	(1)	(2)	respeff3
27.	I feel that I am not in control of my lung/respiratory problem:	(1)	(2)	respeff4
28.	I do not expect my lung/respiratory problem to get any better:	(1)	(2)	respeff5
29.	I have become frail or an invalid because of my lung/respiratory problem:	(1)	(2)	respeff6
30.	Exercise is not safe for me:	(1)	(2)	respeff7
31.	Everything seems too much of an effort:	(1)	(2)	respeff8

ID Number:	0	0		-					-		
Letter Code:											
Form Type:	R	Q	0	1							

Section 5: These are questions about your lung/respiratory medication, including oxygen, inhalers and pills. If you are not receiving medications, go to Section 6. To complete this section, check either True or False as it applies to you now.

		True	False	
32.	My lung/respiratory medication does not help me very much:	(1)	(2)	resprx1
33.	I get embarrassed using my lung/respiratory medication in public:	(1)	(2)	resprx2
34.	I have unpleasant side effects from my lung/respiratory medication:	(1)	(2)	resprx3
35.	My lung/respiratory medication interferes with my life a lot:	(1)	(2)	resprx4

Section 6: These are questions about how your activities might be affected by your breathing problem. For each question, please check True if one or more parts applies to you because of your breathing problem. Otherwise, check False.

		True	False	
36.	I take a long time to get washed or dressed:	(1)	(2)	respact1
37.	I cannot take a bath or shower, or I take a long time:	(1)	(2)	respact2
38.	I walk slower than other people my age, or I stop to rest:	(1)	(2)	respact3
39.	Jobs such as household chores take a long time, or I have to stop to rest:	(1)	(2)	respact4
40.	If I walk up one flight of stairs, I have to go slowly or stop:	(1)	(2)	respact5
41.	If I hurry or walk fast, I have to stop or slow down:	(1)	(2)	respact6
42.	My breathing makes it difficult to do things such as walking up hills, carrying things up stairs, light gardening such as weeding, dancing, playing golf, or light sports such as horseshoes:	(1)	(2)	respact7
43.	My breathing problem makes it difficult to do things such as carrying heavy loads, like digging in the garden or shoveling snow, jogging or walking briskly, playing tennis or swimming:	(1)	(2)	respact8
44.	My breathing problem makes it difficult to do things such as very heavy manual labor, riding a bike, running, swimming fast or playing competitive sports:	(1)	(2)	respact9

ID Number:	0	0		-					-	
Letter Code:										
Form Type:	R	Q	0	1						

Section 7: We would like to know how your breathing usually affects your daily life. Please check either True or False as it applies to you because of your lung/respiratory problem. (Remember that True only applies to you if you can not do something because of your lung/respiratory problem).

		True	False	
45.	I cannot play sports or active games:	(1)	(2)	lmtact1
46.	I cannot go out for entertainment or recreation:	(1)	(2)	lmtact2
47.	I cannot go out of the house to do the grocery shopping:	(1)	(2)	lmtact3
48.	I cannot do household chores:	(1)	(2)	lmtact4
49.	I cannot move far from my bed or chair:	(1)	(2)	lmtact5

50. Here is a list of other activities that your lung/respiratory problem may prevent you from doing. (You do not have to check these, they are just to remind you of ways in which your shortness of breath may affect you):

Going for walks or walking the dog.
Doing activities or chores at home or in the garden.
Having sexual intercourse.
Going to church, or a place of entertainment.
Going out in bad weather or into smoky rooms.
Visiting family or friends or playing with children.

Please write in any other important activities that your lung/respiratory problem may stop you from doing:

a. ______________________________ lmta_sp

b. ______________________________ lmtb_sp

c. ______________________________ lmtc_sp

51. Now, would you check (one only) which you think best describes how your breathing problem affects you. respprb

It does not stop me from doing anything I would like to do:	(1)
It stops me from doing one or two things I would like to do:	(2)
It stops me from doing most of the things I would like to do:	(3)
It stops me from doing everything I would like to do:	(4)

Thank you for filling in this questionnaire. Before you finish, please check to see that you have answered all of the questions.

ID Number:	0	0		-					-	
Letter Code:										
Form Type:	R	Q	0	1						

II. ADMINSTRATIVE MATTERS

1. General Comments: ______________________________ gen_cmnt

____

2. Research Coordinator:

A. Signature: ______________________________ cert_sig

B. LTRC Staff No. ___ ___ ___ - ___ ___ ___ cert_no

3. Date Form Completed: ___ ___ ___ - ___ ___ - 2 ___ ___ ___ compl_dt
Month Day Year

LUNG TISSUE RESEARCH CONTORTIUM (LTRC)
ENVIRONMENTAL QUESTIONNAIRE

ID Number:	0	0		-					-	
Letter Code:										
Form Type:	E	Q	0	1						

1. Date of Interview: ___ ___ ___ - ___ ___ - 2 ___ ___ ___ vis_dt
Month Day Year

I. HOUSEHOLD CHARACTERISTICS

Now, I want to ask some questions about the house(s) you have lived in. As we talk about these conditions or exposures, please tell me if you have been exposed to these conditions and how long you were exposed to these conditions. We are looking for total exposure, so if you had an exposure for six months in one period and an exposure of eight months in another period, your total exposure would be for about one year. Respond to seasonal exposures as if they were for a full year even if the exposure was for a few months (e.g., swimming).

IF PARTICIPANT ANSWERS "NO" or "DON'T KNOW" to EXPOSURE, GO TO THE NEXT ACTIVITY.

		A Exposure			B	
		**Yes**	**No**	**Don't Know**	**Number of Years**	**Don't Know**
1. Have you ever used a wood or coal burning stove or fireplace with an open flame in your home?	heat1	(1)	(2)	(3)	heat1yr ___ ___	(1) heat1dk

2. I'm going to read you a list of devices. For each device, tell me if you ever used it in your home. If you did, tell me for how long you were exposed to it.

		(1) Exposure			(2)	
		**Yes**	**No**	**Don't Know**	**Number of Years**	**Don't Know**
A. Humidifier/Cool Mist Vaporizer	dev1	(1)	(2)	(3)	dev1yr ___ ___	(1) dev1dk
B. Sauna/Hot tub	dev2	(1)	(2)	(3)	dev2yr ___ ___	(1) dev2dk

ID Number:	0	0		-					-	
Letter Code:										
Form Type:	E	Q	0	1						

			(A) Exposure		(B)	
		Yes	No	Don't Know	Number of Years	Don't Know
3.	Did your bathroom(s) or basement ever have visible mold or mildew on indoor surfaces? cndhm	(1)	(2)	(3)	cndhmyr __ __	(1) cndhmdk
4.	Please tell me if you, or anyone living in your house, ever had birds stay <u>inside</u> your home. Please tell me the number of years you've had them. birdhm	(1)	(2)	(3)	birdhmyr __ __	(1) birdhmdk
5.	Please tell me if you ever used pillows with feathers or down and, if you did, for how long you used it. pillow	(1)	(2)	(3)	pillowyr __ __	(1) pillowdk

II. SPECIFIC EXPOSURES CHART

Now I would like to ask some questions that deal with specific materials or substances that have been in the air (as dust, fumes, or vapor) in your JOBS or in your HOBBIES, at work or at home. Wearing these metals in jewelry does not count as an exposure.

> ASK ITEM A FOR EACH MATERIAL LISTED IN THE SPECIFIC EXPOSURES CHART.
>
> A. Have you ever been exposed to (material/substance) as dust or fumes? IF NO OR DON'T KNOW, ASK EXPOSURE (ITEM A) ABOUT NEXT MATERIAL.
>
> B. How long were you exposed to (material/substance)?

				(A) Exposure		(B)	
			Yes	No	Don't Know	Number of Years	Don't Know
1.	Beryllium	expos1	(1)	(2)	(3)	expos1yr __ __	(1) expos1dk
2.	Cobalt	expos2	(1)	(2)	(3)	expos2yr __ __	(1) expos2dk
3.	Asbestos	expos3	(1)	(2)	(3)	expos3yr __ __	(1) expos3dk
4.	Silica	expos4	(1)	(2)	(3)	expos4yr __ __	(1) expos4dk
5.	Arsenic	expos5	(1)	(2)	(3)	expos5yr __ __	(1) expos5dk
6.	Cadmium	expos6	(1)	(2)	(3)	expos6yr __ __	(1) expos6dk

**ID Number:**	0	0		-					-	
**Letter Code:**										
**Form Type:**	E	Q	0	1						

III. ADMINISTRATIVE MATTERS

1. General Comments: ____ gen_cmnt

____

2. Research Coordinator:

A. Signature: ____ cert_sig

B. LTRC Staff Number: __ __ __ - __ __ __ cert_no

3. Date Form Completed: __ __ __ - __ __ - 2 __ __ __ compl_dt
Month Day Year

LUNG TISSUE RESEARCH CONSORTIUM (LTRC)
OCCUPATIONAL AND ENVIRONMENTAL QUESTIONNAIRE

ID Number:	0	0		-					-	
Letter Code:										
Form Type:	O	Q	0	1						

1. Date of Interview: __ __ __ - __ __ - 2 __ __ __ vis_dt
Month Day Year

I. ACTIVITIES ON THE JOB

Now I would like to ask you some questions about specific job related activities. I will read slowly from a long list and ask you whether you have **ever** had a job — even if the job lasted less than six months — that involved any of the following activities. Please tell me if you have worked in any of them and how long, in years, you worked at the job. Please round fractions of years to the nearest whole number.

IF PARTICIPANT ANSWERS "NO", GO TO THE NEXT ACTIVITY.

Ask each activity in turn and pause briefly for each activity. If patient does not answer, check NO and go to the next activity.

ID Number:	0	0		-					-	
Letter Code:										
Form Type:	O	Q	0	1						

		A Employment Yes	No	B Number of Years	Don't Know
1.	Aircraft/aerospace manufacturing	jobacm (1)	(2)	jobacmyr ___ ___	jobacmdk (1)
2.	Animal laboratory worker	jobalw (1)	(2)	jobalwyr ___ ___	jobalwdk (1)
3.	Auto or truck repair	jobatr (1)	(2)	jobatryr ___ ___	jobatrdk (1)
4.	Automotive manufacturing	jobam (1)	(2)	jobamyr ___ ___	jobamdk (1)
5.	Raising birds	jobrb (1)	(2)	jobrbyr ___ ___	jobrbdk (1)
6.	Carpentry or woodworking	jobcww (1)	(2)	jobcwwyr ___ ___	jobcwwdk (1)
7.	Construction	jobcon (1)	(2)	jobconyr ___ ___	jobcondk (1)
8.	Demolition of buildings	jobdob (1)	(2)	jobdobyr ___ ___	jobdobdk (1)
9.	Electrical or electronic worker	jobeew (1)	(2)	jobeewyr ___ ___	jobeewdk (1)
10.	Farming, ranching, farm laborer (wage laborer)	jobfrl (1)	(2)	jobfrlyr ___ ___	jobfrldk (1)
11.	Fire fighter	jobff (1)	(2)	jobffyr ___ ___	jobffdk (1)
12.	In a sawmill	jobsm (1)	(2)	jobsmyr ___ ___	jobsmdk (1)
13.	In a pulpmill	jobpm (1)	(2)	jobpmyr ___ ___	jobpmdk (1)
14.	Hairdressing or cosmetology	jobhc (1)	(2)	jobhcyr ___ ___	jobhcdk (1)
15.	Meat wrapping	jobmw (1)	(2)	jobmwyr ___ ___	jobmwdk (1)
16.	Any type of mining	jobmin (1)	(2)	jobminyr ___ ___	jobmindk (1)
17.	Dentist, dental product maker, or dental technician	jobden (1)	(2)	jobdenyr ___ ___	jobdendk (1)
18.	In plant nursery or as a florist	jobpnf (1)	(2)	jobpnfyr ___ ___	jobpnfdk (1)
19.	Plastics manufacturing	jobplm (1)	(2)	jobplmyr ___ ___	jobplmdk (1)
20.	Working with resins, polyurethane paints, or polyurethane foam manufacturing, or isocyanate paints	jobwwr (1)	(2)	jobwwryr ___ ___	jobwwrdk (1)

ID Number:	0	0		-					-	
Letter Code:										
Form Type:	O	Q	0	1						

		A Employment Yes	A Employment No	B Number of Years	Don't Know
21.	Pottery making or ceramics	jobpmc (1)	(2)	jobpmcyr ___ ___	jobpmcdk (1)
22.	Working in a quarry	jobqua (1)	(2)	jobquayr ___ ___	jobquadk (1)
23.	Sandblasting	jobsb (1)	(2)	jobsbyr ___ ___	jobsbdk (1)
24.	Smelting in a foundry	jobsf (1)	(2)	jobsfyr ___ ___	jobsfdk (1)
25.	Stone cutting or polishing	jobscp (1)	(2)	jobscpyr ___ ___	jobscpdk (1)
26.	Tunnel construction	jobtc (1)	(2)	jobtcyr ___ ___	jobtcdk (1)
27.	Veterinarian/veterinary work	jobvet (1)	(2)	jobvetyr ___ ___	jobvetdk (1)
28.	Welding	jobwld (1)	(2)	jobwldyr ___ ___	jobwlddk (1)
29.	Rubber factory worker	jobrfw (1)	(2)	jobrfwyr ___ ___	jobrfwdk (1)
30.	In a pet store	jobps (1)	(2)	jobpsyr ___ ___	jobpsdk (1)
31.	In an occupation with radiation exposure	jobrde (1)	(2)	jobrdeyr ___ ___	jobrdedk (1)

TELL RESPONDENT "THIS IS THE END OF THE LIST."

32. In your office or indoor working environment, other than in the workplace bathrooms, have you ever noticed any of the following conditions: high humidity; water damage to furnishings, ceiling, tiles, or carpets; obvious mold or mildew not in a bathroom; or musty or moldy odors? **IF YES, SPECIFY DURATION.**

(A) Exposure Yes	(A) Exposure No	(B) Number of Years	(B) Don't Know
wrkenv (1)	(2)	wrkenvyr ___ ___	wrkenvdk (1)

II. ADMINISTRATIVE MATTERS

1. General Comments: ______________________________ gen_cmnt

____

2. Research Coordinator:

A. Signature: ______________________________ cert_sig

B. LTRC Staff Number: ___ ___ ___ - ___ ___ ___ cert_no

3. Date Form Completed: ___ ___ - ___ ___ - 2 ___ ___ ___ compl_dt
Month Day Year

LUNG TISSUE RESEARCH CONSORTIUM (LTRC)
SIX MINUTE WALK TEST

ID Number:	0	0		-					-	
Letter Code:										
Form Type:	W	T								

vis_dt

1. Date of Most Recent Test: ___ ___ ___ - ___ ___ - 2 ___ ___ ___
Month Day Year

I. SIX MINUTE WALK TEST

smw_why

1. Is the test performed as: LTRC Protocol (1) Clinical Care (2)

2. Is resting O_2 saturation at least 88% after appropriate O_2 titration? Yes (1) No (2) ↓ reso2sat

If NO, GO TO PART II.

3. O_2 liter flow at rest: ___ ___ . ___ L/min reso2flo

4. Borg scale rating for perceived breathlessness at rest: restborg ___ ___ . ___ Not Done (1) rborgnd

5. Borg scale rating for leg fatigue at rest: rstborlg ___ ___ . ___ Not Done (1) rlborgnd

6. O_2 liter flow during exercise: ___ ___ . ___ L/min exo2flo

7. Total distance walked

   A. Distance ___ ___ ___ ___ distwalk

   B. Units Meters (1) Feet (2) distunit

8. O_2 saturation at termination: ___ ___ ___ % tero2sat

9. Borg scale rating for perceived breathlessness at termination? termborg ___ ___ . ___ Not Done (1) tborgnd

10. Borg scale rating for leg fatigue at termination? trmborlg ___ ___ . ___ Not Done (1) tlborgnd

11. Reason(s) for test termination ***check "test lasted six minutes" if test terminated at six minutes; otherwise check all that apply of items 10B – 10J.***

   A. Test lasted six minutes (1) term6min
   B. Chest pain: (1) termcp
   C. Near syncope: (1) termsyn
   D. Ataxic gait: (1) termatax
   E. Lower extremity claudication: (1) termclau
   F. Mental confusion: (1) termment

ID Number:	0	0		-					-	
Letter Code:										
Form Type:	W	T								

G. Patient refused to continue: (1) termref
H. Leg Fatigue (1) termleg
I. Staff request: (1) termstaf
J. Other (1) termoth
1) Specify ______________________ term_sp

II. ADMINISTRATIVE MATTERS

1. General Comments: ______________________ gen_cmnt

____

2. Six Minute Walk Tester:

A. Signature: ______________________ test_sig

B. LTRC Staff No. ___ ___ ___ - ___ ___ ___ test_no

3. Research Coordinator:

A. Signature: ______________________ cert_sig

B. LTRC Staff No. ___ ___ ___ - ___ ___ ___ cert_no

4. Date Form Completed: ___ ___ - ___ ___ - 2 ___ ___ ___ compl_dt
Month Day Year

LUNG TISSUE RESEARCH CONSORTIUM (LTRC)
CARDIOPULMONARY EXERCISE TESTING FORM

ID Number:	0	0		-					-	
Letter Code:										
Form Type:	C	E	0	1						

1. Date of Examination: ___ ___ ___ - ___ ___ - 2 ___ ___ ___ (Month, Day, Year) vis_dt

I. PRELIMINARY TEST INFORMATION

1. Is the test performed as: cpx_why
   - LTRC Protocol (1)
   - Clinical Care (2)

2. Best FEV_1: ___ . ___ ___ L bestfev1

FEV_1 x 40 = MVV (L/min)
**If MVV ≤ 40 L/min, then use ramp rate of 5 watts/min**
**If MVV > 40 L/min but < 80 L/min, then use ramp rate of 10 watts/min**
**If MVV ≥ 80 L/min, then use ramp rate of 15 watts/min**

3. Ramp Rate for exercise test: ramprate
   - 5 watts/min (1)
   - 10 watts/min (2)
   - 15 watts/min (3)

ID Number:	0	0		-					-	
Letter Code:										
Form Type:	C	E	0	1						

II. CARDIOPULMONARY EXERCISE TEST

Quantity		A<br>5 Minute Rest	B<br>3 Minute Unloaded	C<br>Maximum
1. Was test completed?		Yes (1) No (2)	Yes (1) No (2)	Yes (1) No (2)
Complete for LEVEL I and II Testing		cmp5mrst	cmp3munl	cmpmax
2. Exercise F_IO2 exerfio2		Room Air (0.21) (1)<br>30% Oxygen (0.30) (2)	N/A	N/A
3. Barometric Pressure (mmHg):	XXX	barpresa	N/A	N/A
4. Equipment Deadspace (cc):	XXX	eqdedspa	N/A	N/A
5. SpO2 (%)	XXX	spo2a	spo2b	spo2c
6. Ve (BTPS; L/min)	XXX.X	vebtpsa	vebtpsb	vebtpsc
7. Vt (BTPS; L)	X.XX	vtbtpsa	vtbtpsb	vtbtpsc
8. VO2 (STPD; L/min)	X.XXX	vo2stpda	vo2stpdb	vo2stpdc
9. VCO2 (STPD; L/min)	X.XXX	vco2a	vco2b	vco2c
10. Heart Rate (beats/minute)	XXX	hrtratea	hrtrateb	hrtratec
11. Respiratory Rate (breaths/minute)	XX	respa	respb	respc
12. Systolic blood pressure (mmHg)	XXX	sbpa	sbpb	sbpc
13. Diastolic blood pressure (mmHg)	XXX	dbpa	dbpb	dbpc
14. Borg (breathlessness)	XX.X	borgbta	borgbtb	borgbtc
15. Borg (leg muscle fatigue)	XX.X	borglga	borglgb	borglgc
16. Load (watts)	XXX	N/A	N/A	loadwtc
Complete for LEVEL II Testing Only				
17. Pa_{O2} (mmHg)	XXX	pao2a	pao2b	pao2c
18. Pa_{CO2} (mmHg)	XXX	paco2a	paco2b	paco2c
19. pH	X.XX	pha	phb	phc
20. Base Excess	XX.X	basexa	basexb	basexc
21. FE_{CO2}	X.XXXX	feco2a	feco2b	feco2c

22. Did the staff member terminate the test? Yes (1) No (2) stterm

If NO, go to Question 23.

Reason staff member terminated the test session. (Check all that apply)

A. Cadence dropped below 40 rpm and did not return (1) stcad
B. Mental confusion (1) stment
C. EKG arrhythmia (1) starrhy
D. EKG ischemia (1) stisch
E. Elevated blood pressure (1) stelevbp
F. Low blood pressure (1) stlowbp
G. Other (1) stothr
1) Specify ______________________ stoth_sp

ID Number:	0	0		-					-	
Letter Code:										
Form Type:	C	E	0	1						

23. Did the patient terminate the test? Yes (1) No (2) ptterm

If NO, go to Part III.

Reason patient terminated the test session (Check all that apply):

A. Dyspnea or shortness of breath (1) ptdysp
B. Dizziness or lightheadedness (1) ptdizz
C. Chest pain (1) ptchtpn
D. Leg fatigue (1) ptleg
E. Other (1) ptothr
   1) Specify ________________ ptoth_sp

III. ADMINISTRATIVE MATTERS

1. General Comments: ________________ gen_cmnt

____

2. Cardiopulmonary Exercise Tester:

   A. Signature: ________________ cpex_sig

   B. LTRC Staff Number: ___ ___ ___ - ___ ___ ___ cpex_no

3. Research Coordinator:

   A. Signature: ________________ cert_sig

   B. LTRC Staff Number: ___ ___ ___ - ___ ___ ___ cert_no

4. Date Form Completed: ___ ___ - ___ ___ - 2 ___ ___ ___ compl_dt
   Month Day Year

LUNG TISSUE RESEARCH CONSORTIUM
PULMONARY FUNCTION TESTING

ID Number:	0	0		-					-	
Letter Code:										
Form Type:	P	F								

1. Date of Study Visit: ___ ___ ___ - ___ ___ - 2 ___ ___ ___ vis_dt
Month Day Year

I. DEMOGRAPHIC INFORMATION

1. Height ___ ___ . ___ inches height

A. Height is measured by:

Standing Height (1) Arm Span (2) htmeas

2. Weight ___ ___ ___ pounds weight

3. With which race or ethnicity do you identify? (Check only one). race

A. White (Caucasian) (1)
B. Hispanic (2)
C. African-American (whether Hispanic or not) (3)
D. Asian or Pacific Islander (4)
E. Native American (5)
F. Other, more than one, or none of the above (6)

II. SPIROMETRY

1. Was the test: spir_wh
Done at LTRC Center (1)
Done at Other Institution (2)
Not Done (3)

If Spirometry not done, go to Part III.

A. Date of Spirometry: ___ ___ ___ - ___ ___ - 2 ___ ___ ___ spir_dt
Month Day Year

prbs_nd
2. Pre-Bronchodilator Spirometry not done (1)

IF PRE-BRONCHODILATOR SPIROMETRY NOT DONE, GO TO PART II, ITEM 4.

3. Pre-Bronchodilators

A.	FEV_1:	pre_fev1 ___ . ___ ___ L	Not Done (1)	prfev_nd
B.	FVC:	pre_fvc ___ . ___ ___ L	Not Done (1)	prfvc_nd
C.	FEV_6:	prfev6 ___ . ___ ___ L	Not Done (1)	prfev6nd
D.	PEFR:	prpefr ___ ___ . ___ L/Second	Not Done (1)	prpefrnd

ID Number:	0	0		-					-	
Letter Code:										
Form Type:	P	F								

4. Post-Bronchodilators

*Post-Bronchodilator Spirometry is required if the ratio of FEV_1 to vital capacity is less than 75% or the patient has a clinical indication other than ILD.

A. Post-Bronchodilator Spirometry not done: (1) pobs_nd

IF POST-BRONCHODILATOR SPIROMETRY NOT DONE, GO TO PART III.

B.	FEV_1:	pos_fev1 ___ . ___ ___	L	Not Done	pofev_nd (1)
C.	FVC:	pos_fvc ___ . ___ ___	L	Not Done	pofvc_nd (1)
D.	FEV_6:	pofev6 ___ . ___ ___	L	Not Done	pofev6nd (1)
E.	PEFR:	popefr ___ ___ . ___	L/Second	Not Done	popefrnd (1)
F.*	Vext:	vext ___ . ___ ___ ___	L	Not Done	vext_nd (1)
G.*	$FET_{100\%}$:	fet100 ___ ___ . ___	Second	Not Done	fet100nd (1)

*QC Items

III. LUNG VOLUME

1. Was the test: lungv_wh

Done at LTRC Center	(1)
Done at Other Institution	(2)
Not Done	(3)

IF LUNG VOLUME NOT DONE, GO TO PART IV

A. Date lung volume performed: ___ ___ ___ - ___ ___ - 2 ___ ___ ___ lungv_dt
Month Day Year

2. Technique: Plethysmography (1) Helium Dilution (2) Nitrogen Washout (3) lvtechnq

3.	TLC:	mntlc ___ ___ . ___ ___	L	Not Done	mntlc_nd (1)
4.	Maximum SVC:	maxsvc ___ . ___ ___	L	Not Done	mxsvc_nd (1)
5.	RV:	rv ___ ___ . ___ ___	L	Not Done	rv_nd (1)
6.	Mean FRC:	mnfrc ___ ___ . ___ ___	L	Not Done	mnfrc_nd (1)
7.	Raw-insp	airwy ___ ___ . ___ ___	cm H_2O/Liters/Sec	Not Done	airwy_nd (1)
8.	sGaw-insp	sgaw ___ . ___ ___ ___	L/cm H_2O/Sec/Liter	Not Done	sgaw_nd (1)

ID Number:	0	0		-					-	
Letter Code:										
Form Type:	P	F								

IV. DIFFUSING CAPACITY (D_LCO)

dlco_wh

1. Was the test:
   - Done at LTRC Center (1)
   - Done at Other Institution (2)
   - Not Done (3)

IF D_LCO NOT DONE, GO TO PART V.

dlco_dt

A. Date D_LCO performed: ___ ___ ___ - ___ ___ - 2 ___ ___ ___ (Month, Day, Year)

2. Mean D_LCO (uncorrected for hemoglobin) — mndlco ___ ___ . ___ ml/min/mmHg — Not Done (1) mdlco_nd

3. V_I: — vi ___ . ___ ___ L — Not Done (1) vi_nd

4. V_{ALV} — valv ___ ___ . ___ ___ L — Not Done (1) valv_nd

V. ROOM AIR ARTERIAL BLOOD GAS ANALYSIS (ABG)

artbl_wh

1. Was the test:
   - Done at LTRC Center (1)
   - Done at Other Institution (2)
   - Not Done (3)

IF ABG NOT DONE, GO TO PART VI.

artbl_dt

A. Date of arterial blood draw: ___ ___ ___ - ___ ___ - 2 ___ ___ ___ (Month, Day, Year)

2. PaO_2 — pao2 ___ ___ ___ mmHg — Not Done (1) pao2_nd

3. $PaCO_2$ — paco2 ___ ___ ___ mmHg — Not Done (1) paco2_nd

4. pH: — ph ___ . ___ ___ — Not Done (1) ph_nd

5. O_2 Sat: — o2sat ___ ___ ___ . ___ % — Not Done (1) o2sat_nd

6. COHb: — cohb ___ ___ . ___ gm % — Not Done (1) cohb_nd

**ID Number:**	0	0		-					-	
**Letter Code:**										
**Form Type:**	P	F								

VI. ADMINISTRATIVE MATTERS

gen_cmnt

1. General Comments: ______________________________

____

2. PFT Tester:

   A. Signature: ______________________________ pft_sig

   B. LTRC Staff No. ___ ___ ___ - ___ ___ ___ pft_no

3. Research Coordinator:

   cert_sig

   A. Signature: ______________________________

   B. LTRC Staff No. ___ ___ ___ - ___ ___ ___ cert_no

   compl_dt

4. Date form completed: ___ ___ ___ - ___ ___ - 2 _ _ _
   Month Day Year

LUNG TISSUE RESEARCH CONSORTIUM
LABORATORY DATA FORM

ID Number:	0	0		-					-	
Letter Code:										
Form Type:	L	D								

1. Date of Examination: ___ ___ ___ - ___ ___ - 2 ___ ___ ___ (Month, Day, Year) vis_dt

I. BLOOD TESTS

Complete Question 1 for all patients.

1. CBC

   A. Date of CBC: ___ ___ ___ - ___ ___ - 2 ___ ___ ___ (Month, Day, Year) cbc_dt — (1) Not Done cbc_nd

IF CBC NOT DONE GO TO QUESTION 2.

B. WBC: ___ ___ . ___ X 10^9/L wbc — Not Done (1) wbc_nd

C. WBC Differential: — Not Done (1) diff_nd

1. neutrophilic ___ ___ . ___ ___ % wbcdiff1
2. lymphocytes ___ ___ . ___ ___ % wbcdiff2
3. monocytes ___ ___ . ___ ___ % wbcdiff3
4. eosinophils ___ ___ . ___ ___ % wbcdiff4
5. basophils ___ ___ . ___ ___ % wbcdiff5

D. Hgb: ___ ___ . ___ g/dL hgb — Not Done (1) hgb_nd

E. Hematocrit: ___ ___ . ___ % hemcrt — Not Done (1) hemcr_nd

F. Platelets: ___ ___ ___ . ___ X 10^9/L platlet — Not Done (1) plat_nd

2. LAB CHEMISTRIES

Complete Question 2 if a patient has a clinical indication of ILD. Abstract for non-ILD patient if available.

A. Date of Lab Chemistries: ___ ___ ___ - ___ ___ - 2 ___ ___ ___ (Month, Day, Year) labch_dt — (1) Not Done labch_nd

If Not Done go to Question 3.

B. Rheumatoid Factor (RF): rfcode (1) Present (2) Absent — Not Done (3)

C. Creatine Kinase (CK): ___ ___ ___ ___ U/L ck — Not Done (1) ck_nd

D. Erythrocyte Sedimentation Rate (ESR): ___ ___ ___ mm/hr esr — Not Done (1) esr_nd

ID Number:	0	0		-					-	
Letter Code:										
Form Type:	L	D								

E.	Anti-Nuclear Antibody (ANA):	Positive (1)	Negative (2)	Not Done (3)	ana
F.	Antibodies to double stranded DNA (Anti-dsDNA)	Present (1)	Absent (2)	Not Done (3)	dsdnacod
G.	Jo-1 Antigen	Positive (1)	Negative (2)	Not Done (3)	jolant
H.	Antibodies to SCL-70	(1)	(2)	(3)	anscl70
I.	Antibodies to SS-A	(1)	(2)	(3)	anssa
J.	Antibodies to SS-B	(1)	(2)	(3)	anssb
K.	Anti-centromere Antibodies	(1)	(2)	(3)	ancentro
L.	Extractable Nuclear Antigen (ENA)	(1)	(2)	(3)	ena

3. OTHER CHEMISTRIES

Required for non-ILD patient. Abstract for ILD patient if available.

A. Alpha-1 Antitrypsin Level alalevel ___ ___ ___ mg/dL Not Done (1) alalv_nd

II. ADMINISTRATIVE MATTERS

1. General Comments: ______________________________ gen_cmnt

____

2. Research Coordinator:

A. Signature: ______________________________ cert_sig

B. LTRC Staff No. ___ ___ ___ - ___ ___ ___ cert_no

3. Date Form Completed: ___ ___ - ___ ___ - 2 ___ ___ ___ compl_dt
Month Day Year

LUNG TISSUE RESEARCH CONSORTIUM (LTRC)
TISSUE COLLECTION MAILING FORM

ID Number:	0	0		-					-	
Letter Code:										
Form Type:	T	C	0	1						

The Collection Time is the time when the tissue is first excised from the lung.

1. A. Collection Date of tissue specimens: ___ ___ ___ - ___ ___ - 2 . . . ___ vis_dt
Month Day Year

coltm_hr coltm_mn
B. Collection Time of tissue specimens: ___ ___ : ___ ___
Hr Min

I. SHIPPING INFORMATION

This mailing form must be completed for each tissue specimen that is collected to be shipped to LTRC at the University of Colorado HSC. A shipping list to be sent with the tissue will be generated when data entry of this form is complete.

1. Tissue ID Number (affix barcode label here): ______________________ tissnum

2. Type of tissue submitted for this case: (check one) tisstyp

Lung explant, single	(1)
Lung explant, bilateral	(2)
Lung lobectomy/wedge resection	(3)
Lung biopsy	(4)
LVRS	(5)

3. Lobes sampled, check all that apply:

A.	Left Upper	(1)	samplu
B.	Lingula	(1)	samplg
C.	Left Lower	(1)	sampll
D.	Right Upper	(1)	sampru
E.	Right Middle	(1)	samprm
F.	Right Lower	(1)	samprl

ID Number:	0	0		-					-	
Letter Code:										
Form Type:	T	C	0	1						

The Fixative Completion Time is the time when the last specimen is fixed/frozen.

4. A. Fixative Completion Date: ___ ___ ___ - ___ ___ - 2 ___ ___ ___ fix_dt
Month Day Year

B. Fixative Completion Time: fixtm_hr ___ ___ : fixtm_mn ___ ___
Hr Min

5. Specimens submitted:

		# of Containers with Tissue Shipped to TCL*
A.	Freezing suture line(s) (8 ml tube, biopsy only)	tspec_a
B.	Formalin-fixed, strips (100 ml specimen containers)	tspec_b
C.	HOPE-fixed (25 ml tubes for biopsy, 70 ml tubes for explant)	tspec_c
D.	RNAlater (25 ml tubes for biopsy, 70 ml tubes for explant)	tspec_d
E.	Flash frozen (25 ml tubes, biopsy only)	tspec_e
F.	Flash frozen – 2.0 x 0.5 x length of lobe-strips (explant only)	tspec_f
G.	Glutaraldehyde (8 ml tubes, Mayo UCHSC only)	tspec_g

* Please return all containers to TCL regardless of whether they contain tissue or not.

6. Date samples shipped to TCL: ___ ___ ___ - ___ ___ - 2 ___ ___ ___ ship_dt
Month Day Year

II. ADMINISTRATIVE MATTERS

1. General Comments: ________________________________ gen_cmnt

____

2. Shipped by:

A. Signature: ________________________________ cert_sig

B. Telephone Number: (___ ___ ___) ___ ___ ___ - ___ ___ ___ ___ shiptel

C. LTRC Staff No. ___ ___ ___ - ___ ___ ___ cert_no

3. Date Form Completed: ___ ___ ___ - ___ ___ - 2 ___ ___ ___ compl_dt
Month Day Year

CALL TCL AT (303) 315-5475 TO NOTIFY THEM THAT A SHIPMENT WILL BE SENT VIA FEDEX OR UPS AND PROVIDE THEM WITH THE TRACKING NUMBER(S). SHIP ONLY ON MONDAY TUESDAY, OR WEDNESDAY. DO NOT SEND THIS FORM TO THE TCL.

LUNG TISSUE RESEARCH CONSORTIUM (LTRC)
BLOOD COLLECTION MAILING FORM

ID Number:	0	0		-					-	
Letter Code:										
Form Type:	B	C								

1. Collection Date of Blood Specimen: ___ ___ ___ - ___ ___ - 2 ___ ___ ___ vis_dt
Month Day Year

I. SHIPPING INFORMATION:

THIS MAILING FORM MUST BE COMPLETED FOR EACH BLOOD SPECIMEN THAT IS COLLECTED TO BE SHIPPED TO THE LTRC AT THE UNIVERSITY OF COLORADO HSC. A SHIPPING LIST TO BE SENT WITH THE BLOOD WILL BE GENERATED WHEN DATA ENTRY OF THIS FORM IS COMPLETE.

1. Blood ID Number (affix barcode label here): ______________________ bldnum

2. Specimens submitted:

		# of Containers with Blood Specimens Submitted to TCL*
A.	Blood (Red/Gray Tigertop)	bldspec1
B.	Blood (Gray/Green Tigertop)	bldspec2
C.	Blood (Blue Top)	bldspec3

*Please return all containers to the TCL regardless of whether or not they contain blood specimens.

3. Date samples shipped to TCL: ___ ___ ___ - ___ ___ - 2 ___ ___ ___ blshp_dt
Month Day Year

II. ADMINISTRATIVE MATTERS

1. General Comments: ______________________ gen_cmnt

____

2. Shipped by:

A. Signature: ______________________ cert_sig

B. Telephone Number: (___ ___ ___) ___ ___ ___ - ___ ___ ___ ___ bshptel

C. LTRC Staff Number: ___ ___ ___ - ___ ___ ___ cert_no

3. Date form completed: ___ ___ ___ - ___ ___ - 2 ___ ___ ___ compl_dt
Month Day Year

CALL TCL AT (303) 315-5475 TO NOTIFY THEM THAT A SHIPMENT WILL BE SENT VIA FEDEX OR UPS AND PROVIDE THEM WITH THE TRACKING NUMBER(S). SHIP ONLY ON MONDAY, TUESDAY, OR WEDNESDAY. DO NOT SEND THIS FORM TO THE TCL.

LUNG TISSUE RESEARCH CONSORTIUM (LTRC)
CT SHIPPING RECORD

ID Number:	0	0		-					-	
Letter Code:										
Form Type:	C	S								

1. Date of CT Scan Acquisition: ___ ___ ___ - ___ ___ - 2 ___ ___ ___ vis_dt
Month Day Year

I. SHIPPING INFORMATION

This shipping record must be completed for each CT scan that is collected. A manifest list to be sent with the CT scan to the Mayo Clinic will be generated when data entry of this form is complete.

1. **CT Technologist Input:**

A. Scanner Protocol: scanprot

LTRC Full Three-Phase Protocol (1)
Basic LTRC High-Resolution CT (2)
Retrospective CT obtained at Clinical Center (3)
Retrospective CT from Outside Media (CD etc.) (4)

	1	2	3	4	5	6	7	8
B. Series Number								
C. Number of Images								

2. **Radiologist Input:**

A. Date of CT Scan Interpretation: ___ ___ ___ - ___ ___ - 2 ___ ___ ___ scan_dt
Month Day Year

3. **Study Coordinator Input:**

A. CT Number
(Affix CT label here): ______________________________ ctnum

B. Method of Transfer: Electronic (1) transmeth
Media (2)

C. Date of CT Transfer: ___ ___ ___ - ___ ___ - 2 ___ ___ ___ cttrn_dt
Month Day Year

D. Shipped by: ______________________________ ctshpnam

E. Date of shipment: ___ ___ ___ - ___ ___ - 2 ___ ___ ___ ctshp_dt
Month Day Year

ID Number:	0	0		-					-	
Letter Code:										
Form Type:	C	S								

II. ADMINISTRATIVE MATTERS

1. General Comments: ______________________________ gen_cmnt

   ____

2. CT Technologist:

   A. Name: ______________________________ ct_nam

   B. LTRC Staff Number: ___ ___ ___ - ___ ___ ___ ct_no

3. Study Coordinator:

   A. Signature: ______________________________ cert_sig

   B. LTRC Staff Number: ___ ___ ___ - ___ ___ ___ cert_no

4. Date form completed: ___ ___ ___ - ___ ___ - 2 ___ ___ ___ compl_dt
   Month

Use only a postage-paid pre-addressed envelope and packaging label supplied by the RCL. If you encounter any problems, please contact either of the following:

Julie Buenger
Phone: (507) 266-3575
Fax: (507) 538-7076

Kathleen Mieras
Phone: (507) 284-9187
Fax: (507) 538-0593

Do not send this form to the RCL

LUNG TISSUE RESEARCH CONSORTIUM (LTRC)
CT SCAN REPORT

CT Number:						
Form Type:	S	R	0	1		

1. Date of Report: ___ ___ ___ - ___ ___ - 2 ___ ___ ___ (Month, Day, Year) vis_dt

I. QUANTITATIVE IMAGE ANALYSIS RESULTS

1. CT Acceptable for Quantitative Analysis (1) Yes (2) No ctaccept

   A. If NO, then specify reason: ctacc_rs
      (1) Apparently not Full 3-Phase LTRC Protocol
      (2) Other (Specify): ________________ ctacc_sp

If CT not acceptable for quantitative analysis, go to Part II.

2. 3D Image analysis results Not Done (1) imag3dnd

If 3D Analysis not done, then go to Part II.

	Yes	No	
A. Histogram analysis	(1)	(2)	histanl
B. Texture analysis	(1)	(2)	textanl
C. Bronchial branching analysis	(1)	(2)	bronanl

3. Date of Analysis: ___ ___ ___ - ___ ___ - 2 ___ ___ ___ (Month, Day, Year) anl_dt
4. CT Analyst Name: ________________ anl_nam
5. LTRC Staff No.: ___ ___ ___ - ___ ___ ___ anl_no

II. QUALITATIVE IMAGE ASSESSMENT AND DESCRIPTION

1. CT Study Verification and Image Quality

	(1)	(2)	(3)	(4)	(5)	(6)	(7)	(8)
A. Series Number								
B. Description*								
C. Image Count								
D. Image Quality Assessment**								
E. QA Problem Type***								

*Description Popup Selections: 1) Scout, 2) Inspiration Supine LTRC volumetric scan, 3) Expiration Supine LTRC volumetric scan, 4) Inspiration Prone LTRC volumetric scan, 5) Inspiration HRCT, 6) Expiration HRCT, 7) Prone HRCT, 8) Other Chest CT, 9) Other CT (non-chest scan), 10) Other Chest CT and other CT (non-chest scan), 11) Image Not Viewable, (12) Other Non-CT Scan Data

** Image Quality Selections: 1) Optimal, 2) Good, 3) Fair, 4) Poor, 5) Not Assessable

*** QA Problem Types: 1) Respiratory Motion Artifact, 2) Other Patient Motion Artifact, 3) Grainy/Noisy Images 4) Incorrect LTRC protocol parameters, 5) Other Scanner Artifacts

CT Number:						
Form Type:	S	R	0	1		

2. Specific Regional Findings

LUNG Lobe Regional Distribution	(1) RIGHT						(2) LEFT					
	Upper a		Middle b		Lower c		Upper (X-Lingula) a		Lingula b		Lower c	
	(1) Central	(2) Peripheral	(1) Central	(2) Peripheral	(1) Central	(2) Peripheral	(1) Central	(2) Peripheral	(1) Central	(2) Peripheral	(1) Central	(2) Peripheral
A. Air Trapping	findaruc	findaruc	findarmc	findarmp	findarlc	findarlp	findaluc	findalup	findalgc	findalgp	findallc	findallp
B. Bronchial Thickening	findbruc	findbrup	findbrmc	findbrmp	findbrlc	findbrlp	findbluc	findblup	findblgc	findblgp	findbllc	findbllp
C. Bronchiectasis	findcruc	findcrup	findcrmc	findcrmp	findcrlc	findcrlp	findcluc	findclup	findclgc	findclgp	findcllc	findcllp
D. Bullae	finddruc	finddrup	finddrmc	finddrmp	finddrlc	finddrlp	finddluc	finddlup	finddlgc	finddlgp	finddllc	finddllp
E. Consolidation	finderuc	finderup	findermc	findermp	finderlc	finderlp	findeluc	findelup	findelgc	findelgp	findellc	findellp
F. Crazy Paving Pattern	findfruc	findfrup	findfrmc	findfrmp	findfrlc	findfrlp	findfluc	findflup	findflgc	findflgp	findfllc	findfllp
G. Emphysema	findgruc	findgrup	findgrmc	findgrmp	findgrlc	findgrlp	findgluc	findglup	findglgc	findglgp	findgllc	findgllp
H. Ground Glass Infiltrates	findhruc	findhrup	findhrmc	findhrmp	findhrlc	findhrlp	findhluc	findhlup	findhlgc	findhlgp	findhllc	findhllp
I. Honeycombing	findiruc	findirup	findirmc	findirmp	findirlc	findirlp	findiluc	findilup	findilgc	findilgp	findillc	findillp
J. Micronodules (<5 mm)	findjruc	findjrup	findjrmc	findjrmp	findjrlc	findjrlp	findjluc	findjlup	findjlgc	findjlgp	findjllc	findjllp
K. Mosaic Attenuation	findkruc	findkrup	findkrmc	findkrmp	findkrlc	findkrlp	findkluc	findklup	findklgc	findklgp	findkllc	findkllp
L. Pulmonary Cysts	findlruc	findlrup	findlrmc	findlrmp	findlrlc	findlrlp	findlluc	findllup	findllgc	findllgp	findlllc	findlllp
M. Reticular Infiltrates	findmruc	findmrup	findmrmc	findmrmp	findmrlc	findmrlp	findmluc	findmlup	findmlgc	findmlgp	findmllc	findmllp
N. Septal Thickening	findnruc	findnrup	findnrmc	findnrmp	findnrlc	findnrlp	findnluc	findnlup	findnlgc	findnlgp	findnllc	findnllp
O. Tree in Bud Pattern	findoruc	findorup	findormc	findormp	findorlc	findorlp	findoluc	findolup	findolgc	findolgp	findollc	findollp

Fill in with numbers:

- 0 – Normal/None
- 1 – Mild (1-25% involvement)
- 2 – Moderate (26-50% involvement)
- 3 – Marked (50-75% involvement)
- 4 – Severe (>75% involvement)
- 6 – Region cannot be evaluated / no data available

CT Number:						
Form Type:	S	R	0	1		

3. Ancillary Findings (Check if present)	(1) Right	(2) Left
A. Evidence for prior Thoracic Surgery (Not CABG)	prsurgr	prsurgl
B. Pulmonary Nodules or Masses (>10mm)	pnodr	pnodl
C. Parenchymal Bands	parbndr	parbndl
D. Giant Bulla (at least 1/3 volume of the lungs	gbullar	gbullal
E. Pulmonary Cavities (thick walled)	pulcavr	pulcavl
F. Lobar or segmental collapse	lobcolr	lobcoll
G. Mediastinal/Hilar Mass/Adenopathy	medmasr	medmasl
H. Enlarged Pulmonary Arteries	pulartr	pulartl
I. Pleural Thickening	pleuthr	pleuthl
J. Pleural Effusion	pleuefr	pleuefl
K. Pleural Calcifications	pleucar	pleucal
L. Skeletal Deformity (scoliosis, kyphosis, compression fractures)	skdefr	skdefl
M. Other/Comments (e.g. esophageal dilatation, microlithiasis, etc. ...specify)<br>Specify: ____________________	ctoth_sp	

	(1) Right Image Number(s)	(2) Left Image Number(s)
N. Nodules	nodr_sp	nodl_sp

4. Radiologist Summary Descriptions:

A. Best description of axial distribution of emphysema — ademphys
- (1) None
- (2) Peripheral/Subpleural
- (3) Central/Axial
- (4) Evenly Distributed

B. Best description of craniocaudal distribution of emphysema — cdemphys
- (1) None
- (2) Upper lung predominant
- (3) Lower lung predominant
- (4) Diffuse
- (5) Superior segments of lower lobes predominantly involved

C. Best description of axial distribution of interstitial process — adintprc
- (1) None
- (2) Peripheral/Subpleural
- (3) Central/Axial
- (4) Evenly distributed

D. Best description of craniocaudal distribution of interstitial process — cdintprc
- (1) None
- (2) Upper lung predominant
- (3) Lower lung predominant
- (4) Diffuse

CT Number:						
Form Type:	S	R	0	1		

		A. Primary	B. Secondary	C. Secondary	D. Secondary
5.	Radiologist Diagnosis	ctdiag1	ctdiag2	ctdiag3	ctdiag4

Please fill in a primary diagnosis and up to 3 secondary diagnoses from the Diagnosis List.

Diagnosis List

(1) Centrilobular Emphysema
(2) Panlobular Emphysema
(3) Paraseptal Emphysema
(4) Idiopathic Pulmonary Fibrosis/Usual Interstitial Pneumonia
(5) Nonspecific Interstitial Pneumonia
(6) Desquamative Interstitial Pneumonia
(7) Respiratory Bronchiolitis
(8) Respiratory Bronchiolitis-Associated Interstitial Lung Disease
(9) Lymphocytic Interstitial Pneumonitis
(10) Cryptogenic Organizing Pneumonia / Bronchiolitis Obliterans Organizing Pneumonia
(11) Acute Interstitial Pneumonia / Diffuse Alveolar Damage
(12) Non-Diagnostic
(13) Fibrosis-Uncharacterized
(14) Primary Bronchogenic Carcinoma
(15) Lymphoma
(16) Pulmonary Metastases
(17) Sarcoidosis
(18) Berylliosis
(19) Hypersensitivity Pneumonitis (Acute/Cellular)
(20) Hypersensitivity Pneumonitis (Chronic/Fibrotic)
(21) Autoimmune Disease: Connective Tissue-related (Scleroderma/SLE/Sjogren/PM/DM/MCTD/UCTD)
(22) Bronchiolitis Obliterans / Bronchiolitis
(23) Vasculitis /Capillaritis / Wegener's Granulomatosis
(24) Eosinophilic Granuloma / Langerhans' Cell Granulomatosis / EC
(25) Eosinophilic Pneumonia
(26) Granulomatous Infection: Mycobacterium Tuberculosis
(27) Granulomatous Infection: Atypical Mycobacterium / MAI / etc.
(28) Granulomatous Infection: Fungi
(29) Granulomatous Inflammation Not Otherwise Specified (NOS)
(30) Normal
(50) Other

Specify:
A. ICD-9 ___ ___ ___ . ___ ___ cdiag1sp
B. ICD-9 ___ ___ ___ . ___ ___ cdiag2sp
C. ICD-9 ___ ___ ___ . ___ ___ cdiag3sp
D. ICD-9 ___ ___ ___ . ___ ___ cdiag4sp

CT Number:						
Form Type:	S	R	0	1		

III. ADMINISTRATIVE MATTERS

1. General Comments: ______________________________ gen_cmnt

____

2. Core Lab Radiologist:

   A. Signature: ______________________________ cert_sig

   B. LTRC Staff No.: ___ ___ ___ - ___ ___ ___ cert_no

3. Date form completed: ___ ___ ___ - ___ ___ - 2 ___ ___ ___ compl_dt

   Month Day Year

LUNG TISSUE RESEARCH CONSORTIUM (LTRC)
CT SCAN REPORT SUPPLEMENT FORM

CT Number:						
Form Type:	S	R	0	1		

II. QUALITATIVE IMAGE ASSESSMENT AND DESCRIPTION

1. CT Study Verification and Image Quality

† Sequential number should be entered into the cells starting with 9.

Sequential Number †								
A. Series Number	serno							
B. Description*	imdesc							
C. Image Count	imcnt							
D. Image Quality Assessment**	imqual							
E. QA Problem Type***	imqprb							

*Description Popup Selections: 1) Scout, 2) Inspiration Supine LTRC volumetric scan, 3) Expiration Supine LTRC volumetric scan, 4) Inspiration Prone LTRC volumetric scan, 5) Inspiration HRCT, 6) Expiration HRCT, 7) Prone HRCT, 8) Other Chest CT, 9) Other CT (non-chest scan), 10) Other Chest CT and other CT (non-chest scan), 11) Image Not Viewable, (12) Other Non-CT Scan Data

** Image Quality Selections: 1) Optimal, 2) Good, 3) Fair, 4) Poor, 5) Not Assessable

*** QA Problem Types: 1) Respiratory Motion Artifact, 2) Other Patient Motion Artifact, 3) Grainy/Noisy Images 4) Incorrect LTRC protocol parameters, 5) Other Scanner Artifacts

LUNG TISSUE RESEARCH CONSORTIUM (LTRC)
CENTRAL PATHOLOGY REPORT

**Specimen Number:**						
**Form Type:**	C	P	0	1		

vis_dt

1. Date specimen obtained: ___ ___ ___ - ___ ___ - 2 ___ . _ ___
Month Day Year

Specimen Number:						
Form Type:	C	P	0	1		

I. EVALUATION

1. Final pathologic diagnosis:

		(1) Primary		(2) Secondary		(3) Secondary		(4) Secondary	
		a. LTRC	b. ICD-9	a. LTRC	b. ICD-9	a. LTRC	b. ICD-9	a. LTRC	b. ICD-9
A.	Overall	cpal1	cpai1	cpal2	cpai2	cpal3	cpai3	cpal4	cpai4
B.	Right Upper	cprul1	cprui1	cprul2	cprui2	cprul3	cprui3	cprul4	cprui4
C.	Right Middle	cprml1	cprmi1	cprml2	cprmi2	cprml3	cprmi3	cprml4	cprmi4
D.	Right Lower	cprll1	cprli1	cprll2	cprli2	cprll3	cprli3	cprll4	cprli4
E.	Left Upper	cplul1	cplui1	cplul2	cplui2	cplul3	cplui3	cplul4	cplui4
F.	Lingula	cplgl1	cplgi1	cplgl2	cplgi2	cplgl3	cplgi3	cplgl4	cplgi4
G.	Left Lower	cplll1	cplli1	cplll2	cplli2	cplll3	cplli3	cplll4	cplli4

Please select 1 primary diagnosis and up to 3 secondary diagnoses. Fill in with the following LTRC codes or if "Other" code, specify the ICD-9 code in format xxx.xx:

(1) Emphysema, centrilobular
(2) Emphysema, panlobular
(3) Emphysema, paraseptal
(4) Usual interstitial pneumonia (UIP)
(5) Non-specific interstitial pneumonia (NSIP)
(6) Desquamative interstitial pneumonia (DIP)
(7) Respiratory bronchiolitis (RB)
(8) Respiratory bronchiolitis-interstitial lung disease (RB-ILD)
(9) Lymphocytic interstitial pneumonia (LIP)
(10) Organizing Pneumonia (OP)
(11) Diffuse Alveolar Damage (DAD)
(12) Non-Diagnostic
(13) Fibrosis-Uncharacterized
(14) Honeycomb Lung
(15) Carcinoma, non-small cell
(16) Carcinoma, small cell
(17) Lymphoma
(18) Sarcoma
(19) Sarcoid
(20) Berylliosis
(21) Hypersensitivity Pneumonitis (Cellular)
(22) Hypersensitivity Pneumonitis (Fibrotic)
(23) Bronchiolitis (Constrictive)
(24) Bronchiolitis (Proliferative)
(25) Bronchiolitis (Cellular)
(26) Bronchiolitis (Diffuse panbronchiolitis)
(27) Bronchiolitis (Neuroendocrine Cell Hyperplasia)
(28) Vasculitis/Capillaritis
(29) Eosinophilic Granuloma (EG, LCG)
(30) Eosinophilic Pneumonia
(31) Granulomatous Infection (M Tuberculosis)
(32) Granulomatous Infection (Atypical Tuberculosis (MAI))
(33) Granulomatous Infection (Fungi)
(34) Granulomatous Inflammation (NOS)
(35) Normal
(50) Other

Specimen Number:						
Form Type:	C	P	0	1		

II. ADMINISTRATIVE MATTERS

1. General Comments: ______________________________ gen_cmnt

   ____

2. Core Lab Pathologist:

   A. Signature: ______________________________ cert_sig

   B. LTRC Staff No. ___ ___ ___ - ___ ___ ___ cert_no

3. Date Form Completed: ___ ___ ___ - ___ ___ - 2 ___ ___ ___ compl_dt
   Month Day Year

LUNG TISSUE RESEARCH CONSORTIUM (LTRC)
LOCAL PATHOLOGY REPORT

**ID Number:**	0	0		-					-	
**Letter Code:**										
**Form Type:**	L	P	0	1						

vis_dt

1. Date specimen obtained: ___ ___ ___ - ___ ___ - 2 ___ ___ ___
Month Day Year

ID Number:	0	0		-						-		
Letter Code:												
Form Type:	L	P	0	1								

I. EVALUATION

		A. Primary	B. Secondary	C. Secondary	D. Secondary
1.	Final Pathologic Diagnosis	lpdiag1	lpdiag2	lpdiag3	lpdiag4

Please select 1 primary diagnosis and up to 3 secondary diagnoses. Fill in with the following numbers:

(1) Emphysema, centrilobular
(2) Emphysema, panlobular
(3) Emphysema, paraseptal
(4) Usual interstitial pneumonia (UIP)
(5) Non-specific interstitial pneumonia (NSIP)
(6) Desquamative interstitial pneumonia (DIP)
(7) Respiratory bronchiolitis (RB)
(8) Respiratory bronchiolitis-interstitial lung disease (RB-ILD)
(9) Lymphocytic interstitial pneumonia (LIP)
(10) Organizing Pneumonia (OP)
(11) Diffuse Alveolar Damage (DAD)
(12) Non-Diagnostic
(13) Fibrosis-Uncharacterized
(14) Honeycomb Lung
(15) Carcinoma, non-small cell
(16) Carcinoma, small cell
(17) Lymphoma
(18) Sarcoma
(19) Sarcoid
(20) Berylliosis
(21) Hypersensitivity Pneumonitis (Cellular)
(22) Hypersensitivity Pneumonitis (Fibrotic)
(23) Bronchiolitis (Constrictive)
(24) Bronchiolitis (Proliferative)
(25) Bronchiolitis (Cellular)
(26) Bronchiolitis (Diffuse panbronchiolitis)
(27) Bronchiolitis (Neuroendocrine Cell Hyperplasia)
(28) Vasculitis/Capillaritis
(29) Eosinophilic Granuloma (EG, LCG)
(30) Eosinophilic Pneumonia
(31) Granulomatous Infection (M Tuberculosis)
(32) Granulomatous Infection (Atypical Tuberculosis (MAI))
(33) Granulomatous Infection (Fungi)
(34) Granulomatous Inflammation (NOS)
(35) Normal
(50) Other

Specify: A. ICD-9 ___ ___ ___ . ___ ___ ldiag1sp
B. ICD-9 ___ ___ ___ . ___ ___ ldiag2sp
C. ICD-9 ___ ___ ___ . ___ ___ ldiag3sp
D. ICD-9 ___ ___ ___ . ___ ___ ldiag4sp

ID Number:	0	0		-					-	
Letter Code:										
Form Type:	L	P	0	1						

II. ADMINISTRATIVE MATTERS

1. General Comments: ______________________________ gen_cmnt

____

2. Site Pathologist/Principal Investigator or Co-Investigator:

   A. Signature: ______________________________ path_sig

   B. LTRC Staff No. ___ ___ ___ - ___ ___ ___ path_no

3. Research Coordinator:

   A. Signature: ______________________________ cert_sig

   B. LTRC Staff No. ___ ___ ___ - ___ ___ ___ cert_no

4. Date Form Completed: ___ ___ ___ - ___ ___ - 2 ___ ___ ___ compl_dt
   Month Day Year

LUNG TISSUE RESEARCH CONSORTIUM (LTRC)
CLINICAL DIAGNOSIS REPORT

ID Number:	0	0		-					-	
Letter Code:										
Form Type:	C	D	0	1						

vis_dt

1. Date of final diagnosis: ___ ___ ___ - ___ ___ - 2 ___ ___ ___
Month Day Year

ID Number:	0	0		-					-	
Letter Code:										
Form Type:	C	D	0	1						

I. EVALUATION

		A. Primary	B. Secondary	C. Secondary	D. Secondary
1.	Final Clinical Diagnosis	cldiag1	cldiag2	cldiag3	cldiag4

Please select 1 primary diagnosis and up to 3 secondary diagnoses. Fill in with the following numbers:

(1) Emphysema
(2) Idiopathic pulmonary fibrosis (Idiopathic UIP)
(3) NSIP
(4) Desquamative interstitial pneumonia (DIP)
(5) Respiratory bronchiolitis (RB)
(6) Respiratory bronchiolitis-interstitial lung disease (RB-ILD)
(7) Lymphocytic interstitial pneumonia (LIP)
(8) Cryptogenic Organizing Pneumonia (COP)
(9) Acute interstitial pneumonia (AIP)
(10) Fibrosis-Uncharacterized
(11) Carcinoma, non-small cell
(12) Carcinoma, small cell
(13) Lymphoma
(14) Sarcoid
(15) Berylliosis
(16) Hypersensitivity Pneumonitis
(17) Autoimmune Disease (SLE)
(18) Autoimmune Disease (Sjogren)
(19) Autoimmune Disease (RA)
(20) Autoimmune Disease (Scleroderma)
(21) Autoimmune Disease (PM/DM)
(22) Autoimmune Disease (MCTD)
(23) Autoimmune Disease (UCTD)
(24) Bronchiolitis (Constrictive)
(25) Bronchiolitis (Proliferative)
(26) Bronchiolitis (Cellular)
(27) Bronchiolitis (Diffuse panbronchiolitis)
(28) Bronchiolitis (Neuroendocrine Cell Hyperplasia)
(29) Vasculitis/Capillaritis
(30) Eosinophilic Granuloma (EG, LCG)
(31) Eosinophilic Pneumonia
(32) Granulomatous Infection (M Tuberculosis)
(33) Granulomatous Infection (Atypical Tuberculosis (MAI))
(34) Granulomatous Infection (Fungi)
(35) Granulomatous Inflammation (NOS)
(36) Normal
(50) Other

A. ICD-9 ___ ___ ___ . ___ ___ diag1sp
B. ICD-9 ___ ___ ___ . ___ ___ diag2sp
C. ICD-9 ___ ___ ___ . ___ ___ diag3sp
D. ICD-9 ___ ___ ___ . ___ ___ diag4sp

ID Number:	0	0		-					-	
Letter Code:										
Form Type:	C	D	0	1						

2. Has the six-week final diagnosis been referred to the patient's primary care physician? Yes (1) No (2) Not Necessary (3) notifymd

II. ADMINISTRATIVE MATTERS

1. General Comments: ______________________________ gen_cmnt

____

2. Principal or Co-Investigator:

   A. Signature: ______________________________ pi62_sig

   B. LTRC Staff Number: ___ ___ ___ - ___ ___ ___ pi62_no

3. Research Coordinator:

   A. Signature: ______________________________ cert_sig

   B. LTRC Staff Number: ___ ___ ___ - ___ ___ ___ cert_no

4. Date form completed: ___ ___ ___ - ___ ___ - 2 ___ ___ ___ compl_dt
   Month Day Year

LUNG TISSUE RESEARCH CONSORTIUM (LTRC)
ADVERSE EVENT REPORT FORM

ID Number:	0	0		-					-	
Letter Code:										
Form Type:	A	E								

I. ADVERSE EVENT INFORMATION

1. A. Onset Date: __ __ __ - __ __ - 2 __ __ __ (Month, Day, Year) vis_dt

   B. Onset Time: __ __ : __ __ ontm_hr : ontm_mn

2. A. End Date: __ __ __ - __ __ - 2 __ __ __ (Month, Day, Year) end_dt   Ongoing (1) ongoing

   B. End Time: __ __ : __ __ end_hr : end_mn

3. Adverse Event Description: ________________________ ae_desc

   A. Is this a serious adverse event? (1) Yes (2) No ae_ser

   If this is a serious adverse event, please submit descriptive information (Form 71) and MedWatch 3500 to the DCC.

4. Body System(s) Affected

	Yes	No	
A. Neurological	(1)	(2)	aeneuro
B. Cardiovascular	(1)	(2)	aecard
C. Reticuloendothelial	(1)	(2)	aeretend
D. Pulmonary	(1)	(2)	aepulm
E. Digestive	(1)	(2)	aediges
F. Musculoskeletal	(1)	(2)	aemusc
G. Immunology	(1)	(2)	aeimmun
H. Skin	(1)	(2)	aeskin
I. Urogenital	(1)	(2)	aeurogen
J. ENT	(1)	(2)	aeent
K. Metabolic	(1)	(2)	aemetab
L. Nutritional	(1)	(2)	aenutr
M. Endocrine	(1)	(2)	aeendo
N. Other:	(1)	(2)	aeothr
Specify ________________			aeoth_sp

5. Category of Event: aecateg

   Expected (1)
   Not Expected (2)

6. Outcome: aeoutcm

   Resolved (1)
   Ongoing (2)
   Died (3)

ID Number:	0	0		-					-	
Letter Code:										
Form Type:	A	E								

7. Severity of Event:		aesev
Mild	(1)
Moderate	(2)
Severe	(3)

8. Relationship to LTRC Protocol:		aeprot
Not Related	(1)
Unlikely	(2)
Possibly	(3)
Probably	(4)
Definitely	(5)

II. ADMINISTRATIVE MATTERS

1. General Comments: ______________________________ gen_cmnt

____

2. Principal or Co- Investigator (Signature required for SAE's only):

A. Signature: ______________________________ pi70_sig

B. LTRC Staff No. ___ ___ ___ - ___ ___ ___ pi70_no

3. Research Coordinator:

A. Signature: ______________________________ cert_sig

B. LTRC Staff No. ___ ___ ___ - ___ ___ ___ cert_no

4. Date Form Completed: ___ ___ - ___ ___ - 2 ___ ___ ___ compl_dt
Month Day Year
